# Converged DNA Damage Response Renders Human Hepatocellular Carcinoma Sensitive to CDK7 Inhibition

**DOI:** 10.3390/cancers14071714

**Published:** 2022-03-28

**Authors:** Guiqin Xie, Ailin Zhu, Xinbin Gu

**Affiliations:** 1 Department of Oral Pathology, Howard University, 600 W. Street NW, Washington, DC 20059, USA; ailinzhu.md@gmail.com; 2 Cancer Center, Howard University, 600 W. Street NW, Washington, DC 20059, USA

**Keywords:** hepatocellular carcinoma, transcription, MYC, CDK7 inhibitor, THZ1

## Abstract

**Simple Summary:**

Hepatocellular carcinoma (HCC) is the most common type of primary liver cancer. HCC has a dismal five-year mortality estimate of >95%, ranking as the fourth leading cause of cancer-related mortality worldwide. Despite the recent progression in the treatment of HCC with multikinase inhibitors, immunotherapy, and antiangiogenic monoclonal antibodies, among other newly emerging therapeutics, the efficacy has varied among patients, making HCC a high priority for developing novel targeted therapeutic agents. CDK7 has been exploited as a therapeutic target in HCC. In the present study, we demonstrated that HCC cells were highly susceptible to THZ1, a selective covalent CDK7 inhibitor. We further discovered that transcription factor MYC-promoted cell proliferation renders cancer cells hypersensitive to apoptotic cell death with THZ1 treatment. Our findings indicate that targeting CDK7 with THZ1 may be a new plausible strategy for treating HCC, in which MYC plays crucial roles in cell proliferation and tumor growth.

**Abstract:**

Hepatocellular carcinoma (HCC) is a lethal malignancy with high mortality. The inhibition of cyclin-dependent kinase 7 (CDK7) activity has shown therapeutic efficacy in HCC. However, the underlying molecular mechanisms remain elusive. Here, we show that three HCC lines, HepG2, Hep3B, and SK-Hep-1, were highly susceptible to the CDK7 inhibitor THZ1. In mouse models, THZ1 effectively reduced HepG2 tumor growth and tumor weight. THZ1 arrested cell cycle and triggered MYC-related apoptosis in HepG2. To evaluate how MYC protein levels affected THZ1-induced apoptotic cell death, we overexpressed MYC in HepG2 and found that exogenously overexpressed MYC promoted cell cycle progression and increased cells in the S phase. THZ1 drastically engendered the apoptosis of MYC-overexpressing HepG2 cells in the S and G2/M phases. Importantly, transcription-inhibition-induced apoptosis is associated with DNA damage, and exogenous MYC expression further enhanced the THZ1-induced DNA damage response in MYC-overexpressing HepG2 cells. Consistently, in the HepG2 xenografts, THZ1 treatment was associated with DNA-damage-induced cell death. Together, our data indicate that the converged effect of MYC-promoted cell cycle progression and CDK7 inhibition by THZ1 confers the hypersensitivity of HCC to DNA-damage-induced cell death. Our findings may suggest a new therapeutic strategy of THZ1 against HCC.

## 1. Introduction

Hepatocellular carcinoma (HCC), ranking as the fourth leading cause of cancer-related mortality worldwide [1], is one of the most common types of malignant tumors. HCC is not sensitive to either chemotherapy or radiotherapy [2,3], which leaves very limited treatment options for patients with advanced HCC. Since the approval of the multikinase inhibitor sorafenib for treating HCC, it has been the standard of care worldwide. However, the majority of HCC patients relapse, with a five-year survival rate of less than 10% after treatment [4,5]. Recently, emerging immunotherapy, antiangiogenic monoclonal antibodies, as well as new multikinase inhibitors have demonstrated improved clinical efficacy against HCC [6,7,8,9]. Nevertheless, most patients with HCC are still unable to achieve a durable anticancer benefit. The aggressive nature and limited options of effective therapeutics make HCC a high priority for the development of novel therapeutic agents.

The transcription factor c-MYC (MYC) is well-known to control cell cycle progression, proliferation, and apoptosis. In HCC, MYC is often highly expressed. The overexpression of MYC is commonly the result of genomic amplification at 8q24.1, which was detected in up to 70% of viral- and alcohol-related HCC as well as 40–60% of early HCC. However, it is only observed in a small percentage of dysplastic nodules [10,11]. The MYC signature has been linked with the malignant conversion of preneoplastic hepatic lesions [12]. The inactivation of MYC was sufficient to lead to tumor dormancy, and MYC reactivation restored malignancy and invasiveness in the neoplastic cells [13]. Due to the importance of MYC in HCC initiation and progression, it represents a potential protein for a therapeutic target with which to treat HCC. In cancer cells, transcriptional control is disrupted by genetic alterations; many types of tumors depend on active transcriptional regulation [14,15,16,17,18,19]. The dependence on gene transcription of tumors with dysregulated cell cycle progression has been exploited for therapeutic opportunities [17]. Studies on therapeutic interventions targeting gene transcription have also been carried out in HCC [20,21]. It is known that the cyclin-dependent kinases (CDKs) catalyze the phosphorylation of cyclins involved in the progression of cells throughout the cell cycle [22,23,24]. CDK7 functions not only as a cyclin-dependent kinase that directly regulates cell cycle progression [25,26], but also as a component of basal transcription factor TFIIH, which controls gene transcription regulation [15,27,28,29]. The expression of CDK7 has been identified as a target in HCC tumor tissues [30]. Targeting CDK7 induces apoptosis and inhibits the tumor growth of HCC [31,32]. THZ1, a selective covalent CDK7 inhibitor that downregulates the CDK7-mediated phosphorylation of RNA polymerase II (RNAP II) [33], is known to potently inhibit transcription and reduce short-lived proteins such as MYC and cell cycle regulators [34]. Treatment with THZ1 has shown efficacy in several types of tumors, such as human T-cell acute lymphoblastic leukemia [33], neuroblastoma [34], esophageal squamous cell carcinoma [35], breast cancer [14,35], and small-cell lung cancer [36]. However, it remains unclear whether MYC plays an essential role in the response of HCC to CDK7 inhibition and how MYC may render the sensitivity of HCC to treatment with THZ1. 

In the present study, we demonstrated that the converged effect of MYC-promoted cell cycle progression and THZ1-induced CDK7 inhibition makes HCC hypersensitive to DNA-damage-induced cell death. Our studies indicate that targeting gene transcription by CDK7 inhibition could be a new effective strategy against HCC, in which MYC plays crucial roles in tumor initiation and progression.

## 2. Materials and Methods

### 2.1. Cell Culture

The human-HCC-derived cell lines Hep3B (#HB-8064, ATCC, Manassas, VA, USA), HepG2 (#HB-8065, ATCC), and SK-Hep-1 (#HTB-52, ATCC) were cultured in DMEM supplemented with 10% fetal bovine serum (FBS) and a 1% antibiotic-antimycotic solution in 5% CO_2_ at 37 °C in a humidified incubator.

### 2.2. Cell Viability Assay

The HCC cells were plated at 5000/well in 96-well plates and treated with vehicle or THZ1 following a 10-fold serial dilution. Five days later, cell viability was measured using a cell viability assay kit (#ab232855, Abcam, Cambridge, MA, USA). Briefly, a crystal violet staining assay was performed to determine cell viability according to the manufacturer’s instruction. After the removal of the culture medium and gentle washing with a washing solution, a crystal violet staining solution (50 μL) was added to each well and incubated for 20 min at room temperature. After thorough washing, a solubilization solution was added and incubated again for 20 min at room temperature on a benchtop rocker. The optical density (O.D.) of each well was measured at 595 nm. Data were represented as the percentage of viable (attached) cells calculated against the values of the cells treated with vehicle.

### 2.3. Western Blot Analysis

For xenograft tumors, 10–20 mg tumor tissue was used to prepare lysates. HCC cells cultured in 6-well plates were collected and washed with phosphate-buffered saline. Tumor tissues or collected cells were homogenized in a solution with 50 mM Tris buffer, 150 mM NaCl, 1 mM EDTA, 1% NP40, and proteinase/phosphatase inhibitors. After homogenized cells were centrifuged at 13,000 rpm for 5 min, the supernatant was transferred to new Eppendorf tubes. Thirty-five micrograms of cell lysate was used for western blot analysis. The antibodies directed against MYC (ab32072) and H2AX (ab11175) were purchased from Abcam (Cambridge, MA, USA). The antibodies directed against poly (ADP ribose) polymerase 1 (PARP) (#9542), pH2AX (#9718), or beta-actin (#4970) were purchased from Cell Signaling (Beverly, MA, USA). Antibodies against RNAPII carboxy-terminal domain (CTD) p-Ser2 (04-1571), RNAPII CTD p-Ser5 (04-1572), RNAPII CTD p-Ser7 (04-1570), and RNAPII (ab817) were purchased from MilliporeSigma (Burlington, MA, USA). The antibody against RNAPII (sc-17798) was purchased from Santa Cruz (Dallas, TX, USA). Horseradish-peroxidase-conjugated antirabbit IgG was used as a secondary antibody. Specific protein bands were detected using an ECL system. Specific protein band intensities were quantified using ImageJ software.

### 2.4. Flow Cytometric Analysis of Cell Cycle

Cells were seeded into six-well plates at a density of 1–4 × 10^5^ cells/well 24 h before treatment. Cells were treated with vehicle or THZ1 (200 nM) for 8 (SK-Hep-1) or 24 (HepG2 or Hep3B) h. After treatment, cells were washed with ice-cold PBS and then fixed with cold 70% ethanol and kept at −20 °C for at least 2 h. Cells were washed in a PBS buffer and resuspended in a PBS buffer at a density of 1 × 10^6^ cells/mL with 0.5 µg/mL 4′,6-diamidino-2-phenylindole (DAPI, Thermo Fisher Scientific, Waltham, MA, USA). Analysis of the cell cycle phase distribution was performed in a BD flowcytometer (BD Bioscience, San Jose, CA, USA), and the data were analyzed using the ModFit LT 3.0 program (Verity Software House, Topsham, ME, USA). All of the experiments were performed in at least triplicate.

### 2.5. Flow Cytometry Apoptosis Assay

After incubation with vehicle or THZ1 (200 nM) for 24 (SK-Hep-1) or 48 (HepG2 or Hep3B) hours, cells were collected and incubated with annexin V-FITC and propidium iodide (PI) solutions (BD Bioscience, Cat. 556547) in the dark for 15 min before flow cytometry assay. Annexin-V-positive and PI-negative cells as well as annexin-V-positive and PI-positive cells were regarded as apoptotic cells. The percentage of apoptotic cells was analyzed using the FlowJo software (FlowJo LLC, Ashland, OR, USA).

### 2.6. Exogenous Overexpression of MYC in the HepG2 Cells

A negative control lentivirus and a lentivirus expressing MYC were purchased from Genecopoeia (Rockville, MD, USA). HepG2 cells were transduced with lentivirus at a multiplicity of infection of five in the presence of Polybrene (10 μg/mL). Puromycin (2.5 μg/mL) was used to select cells infected with control lentiviruses (HepG2-LV) or MYC-expressing lentiviruses (HepG2-MYC) for 14 days. The selected cells were subsequently seeded into 6-well plates for treatment with vehicle or THZ1 (200 nM). Cells were collected for cell cycle analysis, the apoptotic assay, or Western blot analysis.

### 2.7. Immunohistochemical Analysis

Formalin-fixed paraffin-embedded tumor sections (5 μm) were used for immunohistochemistry using primary antibodies directed against Ki-67 (Thermo Scientific, Fremont, CA, USA; #RB-9043-P0), cleaved caspase-3 (cCaspase-3) (Cell Signaling, #9664), pH2AX (Cell Signaling, #9718), RNAPII CTD p-Ser2 (Abcam, ab5095), and MYC (Abcam, ab32072). Staining was performed by incubation for 5 min with diaminobenzidine (DAB) using a DAB peroxidase substrate kit (Vector Laboratories, Burlingame, CA, USA).

### 2.8. In Vivo Xenograft Tumor Assays

Six-to-eight-week-old NOD SCID gamma (NSG) mice were used for xenograft studies. Five million cells in a volume of 200 μL of medium containing 45% Matrigel basement membrane matrix (BD Biosciences, Cat. 354234) were inoculated subcutaneously into the right flank of mice 2–3 weeks before treatment. Treatment with vehicle or THZ1 started when the median tumor size reached approximately 200 mm^3^. THZ1 was administered via IP injection at a dose of 10 mg/kg/mouse twice daily for 14 days. The tumor sizes were measured daily with a caliper. After 14 days of treatment, mice with tumors were euthanized, and the tumors were dissected for analysis.

### 2.9. Statistical Analysis

Student’s t-test and one-way ANOVA were used for statistical analysis. All data are expressed as the mean ± standard error. All tests were two-sided, and *p* < 0.05 was considered significant, whereas N.S. indicates not significant.

## 3. Results

### 3.1. HCC Cells Are Highly Sensitive to THZ1 Treatment 

It has been shown that the covalent CDK inhibitor THZ1 potently suppressed cell proliferation and tumor growth in several different types of tumors, which include T cell acute lymphoblastic leukemia, small-cell lung cancer, neuroblastoma, and breast cancer [33,34,35,36]. To determine whether THZ1 is effective against HCC, we tested the HCC cell line HepG2; the HepG2 cells were highly sensitive to THZ1. To test whether the response to THZ1 is unique for this cell line, we treated two other HCC lines: SK-Hep-1 and Hep3B. Dose–response cell viability and proliferation experiments in these cells showed that all of the three HCC lines tested were highly sensitive to THZ1 treatment, with an IC50 value of 5.1 nM for SK-Hep-1, 20.3 nM for Hep3B, and 53.8 nM for HepG2, respectively (Supplementary Figure S1A). 

**Figure 1:**
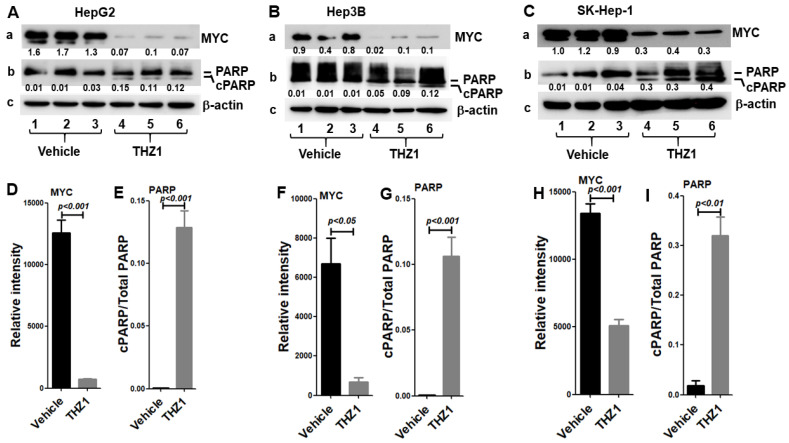
THZ1 decreases MYC expression but increases the cleavage of PARP in HepG2 (**A**), Hep3B (**B**), and Sk-Hep-1 (**C**) cells. Total protein extracts were prepared from the cells treated with vehicle or THZ1 (200 nM). Western blot analysis was performed for MYC (a), PARP (b), and β-actin (c) in HepG2 (**A**), Hep3B (**B**), and SK-Hep-1 (**C**) cells treated with vehicle or THZ1 for 48 h. The numbers below each band are protein band intensities relative to the corresponding β-actin and relative band intensities of cleaved PARP/PARP. (**D**–**I**) Band intensities in (**A**–**C**) were quantified for comparisons between the vehicle-treated cells and the THZ1-treated cells. The data of three independent experiments are presented as mean ± SEM (*n* = 3).

The transcription factor MYC is often highly expressed in HCC, which is known to control cell cycle progression, proliferation, and apoptosis. We examined whether MYC plays a crucial role for THZ1 to inhibit HCC cell proliferation. Using Western blot analysis, we found that the level of MYC protein was reduced to 5.7%, 10.0%, and 37.9% in THZ1-treated cells compared to vehicle-treated cells in Hep3B, HepG2, and SK-Hep-1, respectively (Figure 1A(a),B(a),C(a),D,F,H; Figure S6A–C). Apoptosis is one of the mechanisms with which to control cell proliferation. To examine whether treatment with THZ1 leads to apoptosis, we measured the cleaved PARP (cPARP) protein levels. In the absence of THZ1 treatment, no obvious cleavage of PARP was observed in the Hep3B, HepG2, and SK-Hep-1 cells. However, we found that the cPARP protein levels were markedly increased in THZ1-treated cells compared to vehicle-treated cells in the Hep3B, HepG2, and SK-Hep-1 cell lines (Figure 1A(b),B(b),C(b),E,G,I; Figure S6A–C). These results indicate that MYC acts as a critical mediator for THZ1 to suppress HCC cell proliferation via apoptotic cell death.

### 3.2. THZ1 Exhibits Antitumor Activity in Mouse Model 

**Figure 2:**
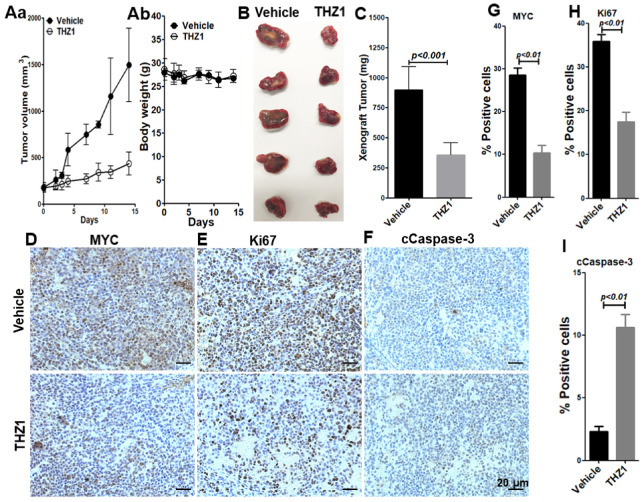
THZ1 inhibits the tumor growth of HepG2 cells in NSG mice. When the tumors reached an average size of 200 mm^3^, vehicle or THZ1 (10 mg/kg/mouse, IP, twice daily) was administered for 14 days. (**A**) Tumor growth (a) and body weight (b) curves plotted for a 14-day treatment period with vehicle or THZ1. (**B**) Images of the dissected tumors from mice treated with vehicle or THZ1. (**C**) Average weight of the dissected tumors from NSG mice after two weeks of treatment. THZ1 treatment reduces MYC (**D**,**G**), reduces Ki67 (**E**,**H**), and increases cCaspase-3 (**F**,**I**) in the HepG2 xenograft tumors. Scale bar = 20 µm. Data are presented as mean ± SEM (*n* = 5).

Since THZ1 treatment effectively reduced MYC and inhibited the proliferation as well as induced the apoptosis of the cultured HCC cells, we next assessed in vivo CDK7 inhibition in a mouse HepG2 xenograft tumor model. We examined the antitumor effects of THZ1 in NSG murine models when subcutaneous tumors reached an average size of 200 mm^3^. We found that THZ1 suppressed the tumor growth (Figure 2A(a)) and reduced the tumor size (Figure 2B) as well as the tumor weight (Figure 2C). However, no significant difference was observed in the body weights (Figure 2A(b)) between the vehicle-treated and THZ1-treated mice during the two weeks of treatment. These in vivo data indicate that THZ1 effectively inhibits the growth of HCC tumors in mice. 

To explore the mechanisms of THZ1-induced tumor growth inhibition, we examined whether THZ1 effectively inhibits MYC in the HepG2 xenograft tumors. With THZ1 treatment, the number of MYC-positive cells was significantly reduced, from 28.5% to 10.4%, indicating that THZ1 reliably inhibits MYC, as we observed in the cultured HepG2 cells (Figure 2D,G). We next measured the proteins crucial for cell proliferation and apoptosis by immunohistochemical analysis. Without THZ1, numerous Ki67-postive cells were found in the HepG2 tumor tissues (Figure 2E). After THZ1 treatment, however, the number of Ki67-positive cells was significantly reduced, from 35.8% to 17.5% in the HepG2 tumor tissues (Figure 2E,H). The number of cCaspase-3-positive cells was significantly increased, from 2.3% in vehicle-treated tumors to 10.6% in THZ1-treated tumors (Figure 2F,I). These results indicate that THZ1 inhibits the in vivo tumor growth of HCC cells via apoptosis, and that MYC may be a critical mediator for the suppression of tumor growth by THZ1.

### 3.3. THZ1 Inhibits Cell Proliferation via Cell Cycle Arrest and Apoptosis Induction 

**Figure 3:**
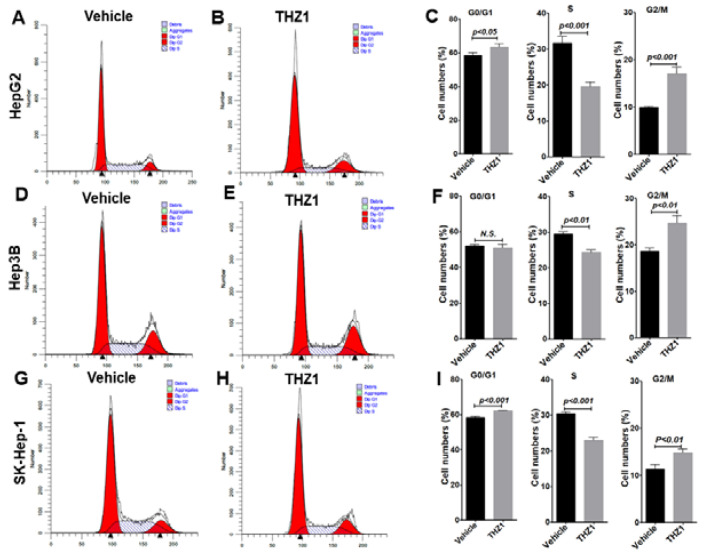
THZ1 induces cell cycle arrest with reduced cell number in the S phase in HepG2 (**A**–**C**), Hep3B (**D**–**F**), and SK-Hep-1 (**G**–**I**) cells. The cells were treated with vehicle or THZ1 (200 nM) for 24 h (HepG2, Hep3B) or 8 h (SK-Hep-1). Flow cytometric analysis of cell cycle phases was carried out after DAPI staining. Data of three independent experiments are presented as mean ± SEM (*n* = 3). N.S. indicates not significant.

Since MYC is critical for cell cycle progression, we examined the effects of THZ1 on the cell cycle in the three HCC lines. In HepG2, THZ1 treatment increased the cells in the G0/G1 and G2/M phases from 58.4% and 9.9% with vehicle treatment to 63.4% and 17.0%, and reduced the cells in the S phase from 31.7% with vehicle treatment to 19.5%, indicating that THZ1 induced G0/G1 and G2/M arrest (Figure 3A–C). In the Hep3B cells treated with THZ1, the cells in the G2/M phases were increased from 18.6% with vehicle treatment to 24.6%, while the cells in the S phase were reduced from 29.4% with vehicle treatment to 24.3%. There was no difference in the G0/G1 phase of the cells with vehicle or THZ1 treatment (Figure 3D–F). Similarly, in the SK-Hep-1 cells treated with THZ1, the cells in the G0/G1 and G2/M phases were increased from 58.3% and 11.3% with vehicle treatment to 62.3% and 14.8%, while the cells in the S phase were reduced from 30.4% with vehicle treatment to 22.9%, (Figure 3G–I). Our observation supports the theory that THZ1 treatment induces a marked reduction in the number of cells in the S phase accompanied with cell cycle arrest in all three HCC cells. 

**Figure 4:**
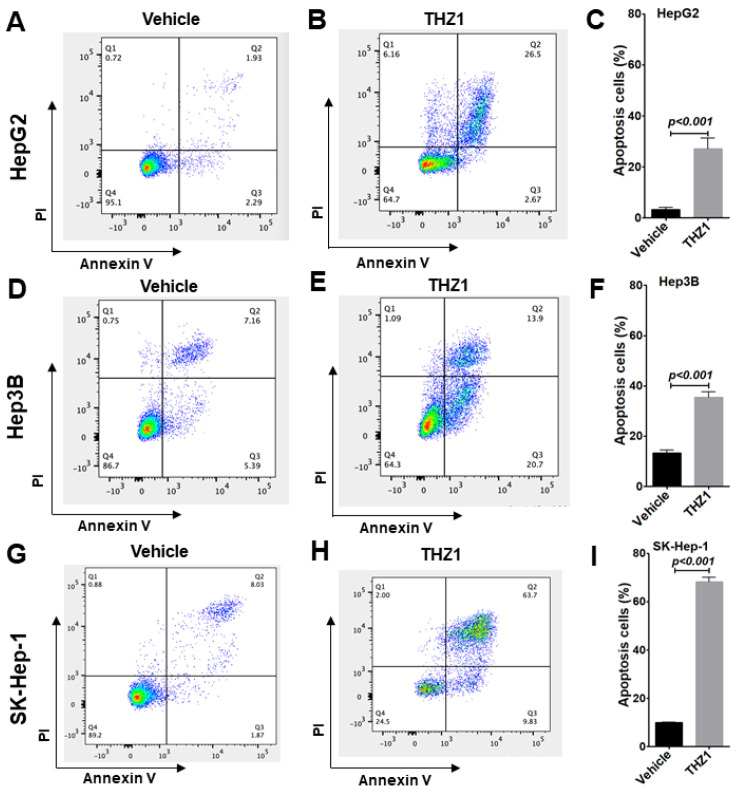
THZ1 induces apoptosis in HepG2 (**A**–**C**), Hep3B (**D**–**F**), and SK-Hep-1 (**G**–**I**) cells. Cells were treated with vehicle or THZ1 (200 nM) for 24 (SK-Hep-1) or 48 (HepG2, Hep3B) h and collected for annexin V as well as PI staining followed by flow cytometric analysis for apoptotic cells. The populations of apoptotic cells were quantified. Data of three independent experiments are presented as mean ± SEM (*n* = 3).

Since the reduction in the number of cells in the S phase accompanied with G2/M arrest is associated with DNA damage and DNA-damage-induced apoptosis, we next evaluated apoptotic events in the three HCC cells. With THZ1 treatment, we found that the apoptotic cells were increased from 3.3%, 13.3%, and 9.9% with vehicle treatment to 27.2%, 35.4%, and 68.3% in HepG2 (Figure 4A–C), Hep3B (Figure 4D–F), and SK-Hep-1 (Figure 4G–I), respectively. 

### 3.4. Overexpressed MYC Promotes Cell Cycle Progression 

As an oncogenic transcription factor, MYC has been found to play pivotal roles in growth control and carcinogenesis [37]. Previously, we showed that, in cells infected with MYC-expressing lentiviruses, the abundance of MYC was found to be increased compared to that of cells infected with control lentiviruses [38]. To assess how THZ1 treatment affects the endogenous MYC and lentiviral MYC expression, we constructed a 3xFLAG-tagged MYC lentiviral expression vector and established a HepG2-3xFLAGMYC cell line (Supplementary Figure S2). We found that the endogenous MYC protein expression was markedly reduced in the THZ1-treated HegG2-LV cells at 6 or 24 h (Supplementary Figure S3A,C), while the lentiviral 3xFLAG-tagged MYC protein expression was not reduced in the THZ1-treated HepG2-3xFLAGMYC cells (Supplementary Figure S3B,D). We also performed allele-specific RT-qPCR to assess the difference in endogenous MYC and lentiviral 3xFLAG-tagged MYC RNA expression in the HepG2-3xFLAGMYC cells treated with vehicle or THZ1. Consistently, we found that the endogenous MYC RNA expression was significantly reduced (Supplementary Figure S4A,B). However, the lentiviral 3xFLAG-tagged MYC RNA expression was not decreased by THZ1 treatment for either 6 or 24 h (Supplementary Figure S4C,D). The differential regulation of endogenous and exogenous MYC may be due to the different promoter that drives MYC expression, while the endogenous MYC is driven by super-enhancers, in which CDK7 is one critical component [20], the exogenously expressed MYC is driven by a viral promoter that is not affected by super-enhancers. 

**Figure 5:**
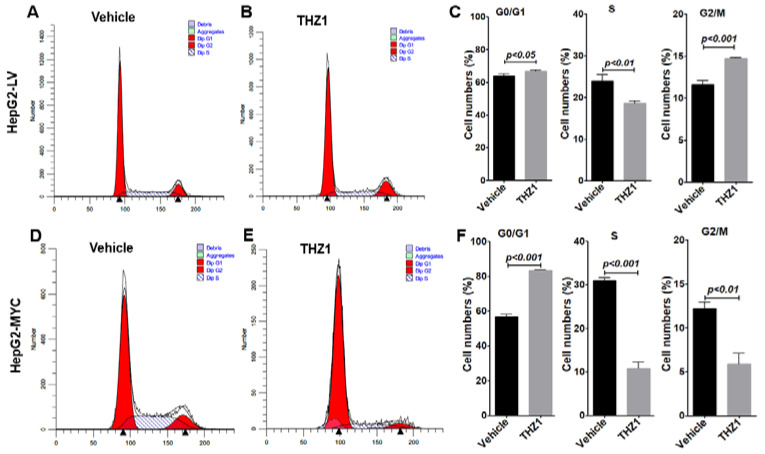
MYC overexpression promotes cell cycle progression and alters response to THZ1. HepG2-LV (**A**–**C**) and HepG2-MYC (**D**–**F**) cells were treated with vehicle or THZ1 (200 nM) for 36 h. A flow cytometric analysis of cell cycle phases was carried out after DAPI staining. Data of three independent experiments are presented as mean ± SEM (*n* = 3).

We then examined whether increased MYC expression is associated with increased cell cycle progression [39] in the HepG2 cells with control or MYC-expressing lentiviruses. In a cell viability assay, THZ1 treatment resulted in a similar IC50 value in HepG2-MYC (51.8 nM) and HepG2-LV (55.2 nM) (Supplementary Figure S1B). We performed a cell cycle analysis after THZ1 treatment for 24 h (Supplementary Figure S5) or 36 h (Figure 5). In the HepG2-LV cells, we observed an increased number of cells in the G2/M phase, from 11.6% with vehicle treatment to 14.8% with THZ1 treatment, indicating a G2/M arrest, as observed in the HepG2 cells (Figure 5A–C). The percentage of cells in the S phase was increased from 23.0% in HepG2-LV to 31.1% in HepG2-MYC, supporting the theory that the elevated expression of MYC promotes cell cycle progression (Figure 5A,C,D,F). Importantly, upon THZ1 treatment, the majority of cells underwent apoptosis. For the remaining vital cells, 83.4% of the vital cells were arrested in the G0/G1 phase, with markedly reduced S and G2/M phases in the HepG2-MYC cells, from 18.6% and 14.8% in the HepG2-LV cells (Figure 5A–C) to 10.7% and 5.9%, respectively (Figure 5D–F). The data support the theory that the HepG2 cells with overexpressed MYC in the S and G2/M phases are highly sensitive to THZ1 treatment for apoptosis. 

### 3.5. Overexpressed MYC Renders Tumor Cells Hypersensitive to THZ1 Treatment

**Figure 6:**
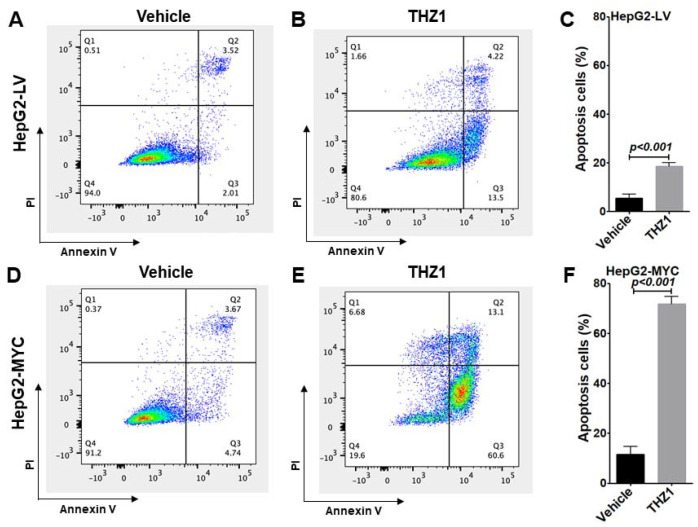
MYC overexpression promotes sensitivity to THZ1-induced apoptosis. HepG2-LV (**A**–**C**) or HepG2-MYC (**D**–**F**) cells were treated with vehicle or THZ1 (200 nM) for 48 h and collected for annexin V and PI staining followed by flow cytometric analysis for apoptotic cells. The populations of apoptotic cells were quantified. Data of three independent experiments are presented as mean ± SEM (*n* = 3).

To validate whether exogenously overexpressed MYC renders the HepG2-MYC cells hypersensitive to CDK7 inhibition, we evaluated THZ1-induced cell death in both the HepG2-LV and HepG2-MYC cells. In the HepG2-LV cells, 5.5% of the cells treated with vehicle were apoptotic cells, while 18.5% of the cells treated with THZ1 were apoptotic cells (Figure 6A–C). In the HepG2-MYC cells, even with vehicle treatment, apoptotic cells were increased from 5.5% in the HepG2-LV control cells (Figure 6A,C) to 8.4% in the MYC-overexpressing cells (Figure 6D,F). Upon THZ1 treatment, THZ1-induced cell apoptosis was markedly increased, from 18.5% in the HepG2-LV control cells (Figure 6B,C) to 71.8% in the MYC-overexpressing cells (Figure 6E,F). Our cell cycle and apoptotic analysis data suggest that MYC-driven cell progression with a markedly increased number of cells in the S and G2/M phases renders HCC with a higher sensitivity to undergo THZ-induced apoptosis. 

### 3.6. THZ1 Reduces RNAP II Phosphorylation

**Figure 7:**
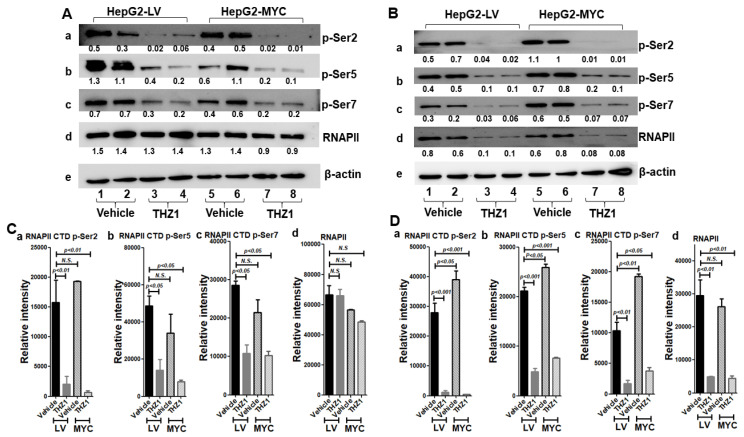
THZ1 inhibits RNAII CTD phosphorylation in HepG2-LV and HepG2-MYC cells. Total protein extracts were prepared from the cells treated with vehicle or THZ1 (200 nM) for 3 h (**A**) or 48 h (**B**). Western blot analysis was performed for RNAPII CTD p-Ser2 (a), RNAPII CTD p-Ser5 (b), RNAPII CTD p-Ser7 (c), RNAPII (d), and β-actin (e). (**C**) Band intensities of p-Ser2 (a), p-Ser5 (b), p-Ser7 (c), and RNAPII (d) in (**A**), and (**D**) band intensities of p-Ser2 (a), p-Ser5 (b), p-Ser7 (c), and RNAPII (d) in (**B**) were quantified for comparisons between vehicle-treated cells and THZ1-treated cells. The relative band intensities of each protein to the corresponding β-actin are shown below the bands. Data of four independent experiments are presented as mean ± SEM (*n* = 4). N.S. indicates not significant.

We examined whether THZ1 suppresses RNAP II carboxy-terminal domain (CTD) phosphorylation in both HepG2-LV and HepG2-MYC cells. We first examined RNAP II CTD phosphorylation after cells were treated with vehicle or THZ1 for 3 h. After THZ1 treatment, the level of phosphorylation at the site of RNAPII CTD p-Ser2, p-Ser5, and p-Ser7 was reduced to 13.0%, 29.2%, and 38.0% of that with vehicle treatment in the HepG2-LV cells; 3.4%, 23.3%, and 47.8% of that with vehicle treatment in the HepG2-MYC cells, respectively (Figure 7A(a–c),C(a–c); Figure S7A). However, RNAPII was not significantly altered in both the HepG2-LV and HepG2-MYC cells (Figure 7A(d),C(d); Figure S7A). There was no significant difference in the levels of phosphorylation at these sites between the HepG2-LV and HepG2-MYC cells (Figure 7A,C). We then examined RNAP II CTD phosphorylation after cells were treated with vehicle or THZ1 for 48 h. After THZ1 treatment, the level of phosphorylation at the site of RNAPII CTD p-Ser2, p-Ser5, and p-Ser7 was reduced to 4.3%, 23.0%, and 16.3% of that with vehicle treatment in the HepG2-LV cells; and 1.3%, 29.4%, and 19.8% of that with vehicle treatment in the HepG2-MYC cells, respectively (Figure 7B(a–c),D(a–c); Figure S7B). There was no significant difference in the levels of phosphorylation at these sites between the HepG2-LV and the HepG2-MYC cells. In addition, the level of RNAPII after THZ1 treatment was reduced to 16.7% and 16.8% of that with vehicle treatment in both the HepG2-LV and HepG2-MYC cells (Figure 7B(d),D(d); Figure S7B). These data suggest that THZ1 effectively inhibits RNAP II CTD phosphorylation in both the HepG2-LV and HepG2-MYC cells, and overexpressed MYC does not affect the inhibition of RNAP II CTD phosphorylation by THZ1, indicating that the differential response of apoptosis is not due to the different sensitivity of the two cell lines to THZ1-induced CDK7 inhibition.

### 3.7. MYC-Conferred Increased DNA Damage Response to THZ1

**Figure 8:**
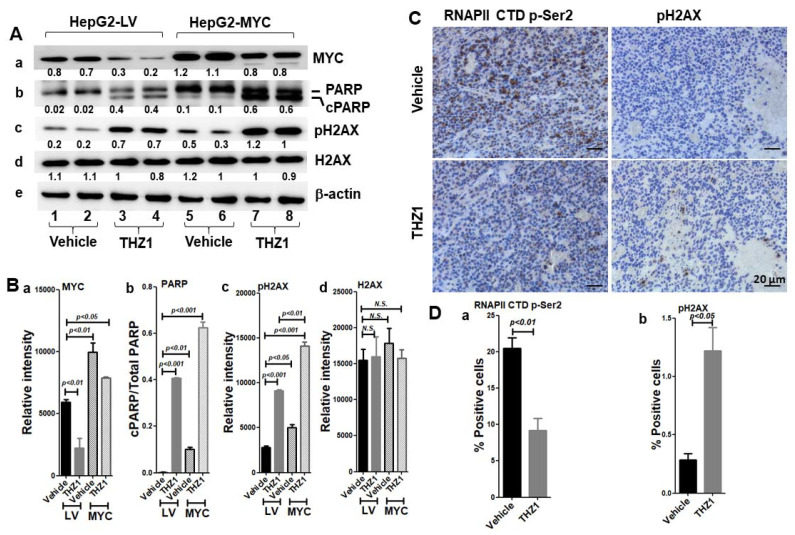
(**A**,**B**) THZ1 reduces MYC and increases the cleavage of PARP and pH2AX in HepG2-LV as well as HepG2-MYC cells. Total protein extracts were prepared from the cells treated with vehicle or THZ1 (200 nM) for 48 h. Western blot analysis was performed for MYC (a), PARP (b), pH2AX (c), H2AX (d), and β-actin (e). (**B**) Band intensities in (**A**) were quantified for comparisons between vehicle-treated cells and THZ1-treated cells. The relative band intensities of each protein to the corresponding β-actin and relative band intensities of cleaved PARP/PARP are shown below the bands. Data of four independent experiments are presented as mean ± SEM (*n* = 4). (**C**,**D**) THZ1 treatment reduces RNAPII CTD p-Ser2 and increases pH2AX in the HepG2 xenograft tumors. (**D**) Relative increases in p-Ser2 (a) and pH2AX (**b**) by THZ1 treatment vs vehicle treatment. Scale bar = 20 µm. Data are presented as mean ± SEM (*n* = 5). N.S. indicates not significant.

To explore the mechanism of THZ1-induced apoptosis and hypersensitivity of the HepG2-MYC cells to THZ1, we examined the MYC protein levels in the HepG2-LV and HepG2-MYC cells. The MYC level in the HepG2-MYC cells with vehicle treatment was 1.7-fold higher than that in the HepG2-LV cells (lanes five–six vs. lanes one–two, Figure 8A(a),B(a); Figure S8A), indicating that 41.2% of MYC was exogenously expressed while 58.5% of MYC was endogenously expressed in HepG2-MYC. In the HepG2-LV cells treated with THZ1, the MYC level decreased by 62.5% of that without THZ1 treatment (lanes three–four vs. lanes one–two, Figure 8A(a),B(a)), and MYC was only reduced by 20.8% in the HepG2-MYC cells (Figure 8A(a),B(a), lanes seven–eight vs. five–six). Importantly, after THZ1 treatment, the MYC level in the HepG2-MYC cells was 1.3-fold and 3.6-fold higher than that in the HepG2-LV cells without or with THZ1 treatment, respectively (lanes seven–eight vs. lanes one–two, lanes seven–eight vs. lanes three–four, Figure 8A(a),B(a)). The data indicate that the decreased level of MYC by THZ1 in the HepG2-MYC cells was due to the reduction in endogenous MYC. 

We then examined the expression of PARP. In the HepG2-LV cells with vehicle treatment, there was no visible cPARP (lanes one–two, Figure 8A(b),B(b); Figure S8B). After THZ1 treatment, the cPARP markedly increased (lanes three–four vs. lanes one–two, Figure 8A(b),B(b)). In contrast, the cPARP was evident in the HepG2-MYC cells even with vehicle treatment (lanes five–six, Figure 8A(b),B(b)); THZ1 further increased the cPARP by 6.2-fold (lanes seven–eight vs. lanes five–six, Figure 8A(b),B(b)). With THZ1 treatment, the cPARP in the HepG2-MYC cells was 1.5-fold higher than that in the HepG2-LV cells (lanes seven–eight vs. lanes three–four, Figure 8A(b),B(b)). The close association of MYC protein expression and cPARP induction indicates that MYC reduction may directly result in THZ1-induced apoptotic cell death in the HepG2 cells with or without MYC overexpression.

It was reported that MYC itself has the capacity to induce apoptosis [40]. It was also reported that THZ1 may induce DNA damage in high-grade glioma [41]. We explored whether THZ1-induced CDK7 inhibition may lead to a DNA damage response. H2AX is required for checkpoint-mediated cell cycle arrest and DNA repair; within minutes following DNA damage, H2AX is phosphorylated at Ser139 at sites of DNA damage [42]. In the HepG2-LV cells treated with THZ1, the level of pH2AX was increased by 3.2-fold (lanes three–four vs. lanes one–two, Figure 8A(c),B(c); Figure S8C). Even in the absence of THZ1, the level of pH2AX in the HepG2-MYC cells was increased by 1.8-fold of that in the HepG2-LV cells (lanes five–six vs. lanes one–two, Figure 8A(c),B(c)). THZ1 further increased the pH2AX level by 2.8-fold (lanes seven–eight vs. lanes five–six, Figure 8A(c),B(c)). With THZ1 treatment, the level of pH2AX in the HepG2-MYC cells was 1.5-fold higher than that in the HepG2-LV cells (lanes seven–eight vs. lanes three–four, Figure 8A(c),B(c)). However, THZ1 treatment did not change the level of H2AX (Figure 8A(d),B(d)). Our results indicate that converged DNA damage due to MYC-promoted cell cycle progression and THZ1-induced CDK7 inhibition accounts for the hypersensitivity of the HepG2 cells to THZ1.

We further examined tissue slices of the vehicle- or THZ1-treated xenograft tumors (Figure 8C,D). THZ1 treatment reduced the level of RNAPII CTD p-Ser2, indicating that THZ1 is effective in inhibiting CDK7 activity in the xenograft tumors (left panel, Figure 8C,D(a)). Importantly, THZ1 resulted in a marked increase in pH2AX by 4.3-fold compared with that in the vehicle-treated tissue (right panel, Figure 8C,D(b)). The pH2AX-positive cells in the xenograft tumor with THZ1 treatment were distributed around the dead tissue foci (lower-right panel, Figure 8C). Taken together, our data support the theory that THZ1 treatment promotes an MYC-mediated apoptotic DNA damage response.

## 4. Discussion

CDK7 has been exploited as a target for the treatment of HCC. We found that THZ1 suppressed the cell proliferation of the Hep3B, HepG2, and SK-Hep-1 cells. These cell lines have numerous genetic alterations with diverse mutational profiles in common oncogenes, i.e., the Hep3B cells are known to carry more than 13 mutations while the HepG2 cells have been reported to carry two mutations in *CTNNB1* (β-catenin) and *NRAS*; the SK-Hep-1 cells carry a *BRAF^V600E^* mutation, as reported by the COSMIC cell lines project. Despite their differences in genetic alterations, THZ1 treatment induced apoptosis in all three cell lines. In the HepG2-MYC cells with exogenously overexpressed MYC, increased MYC expression was strongly correlated to markedly increased DNA damage and apoptosis. In HepG2 tumors, THZ1 induced an apoptotic DNA damage response. Our data support the notion that MYC mediated THZ1-induced apoptotic cell death, the inhibition of cancer cell proliferation, and tumor growth in HCC.

Our study revealed that MYC-driven cell cycle progression renders cancer cells with a higher sensitivity to apoptosis in response to CDK7 inhibition. Since MYC promotes cell cycle progression [39], overexpressed MYC drives more cancer cells into active cell cycle phases. With THZ1 treatment, 71.8% of the HepG2-MYC cells went apoptotic. These apoptotic cells were mainly cancer cells in active S and G2/M phases of the cell cycle. For the remaining vital cells, 83.4% of them were arrested at G0/G1 with markedly reduced cell numbers in the S and G2/M phases (Figure 5D–F). Our observation may well explain a previous report on HCC that the presence of a high level of MYC protein was associated with high sensitivity to CDK7 inhibition, and that exogenous MYC expression in cancer cells with a low level of endogenous MYC led to a greater sensitivity to THZ1 as compared to that of vector control–transfected cells [30].

It was well-documented that the suppression of MYC expression may result in apoptotic cell death. However, in HepG2-MYC cells with a higher level of MYC, we observed even more drastically increased apoptosis (Figure 6). While MYC-reduction-induced apoptosis is well-studied, the mechanism of high MYC-promoted apoptosis is not very well-known. These observations raise the possibility that MYC may affect THZ1-induced apoptosis via distinct mechanisms. Cell cycle analysis indicated that G2 cell cycle arrest by THZ1 itself did not strongly induce cell death in the HepG2-LV cells (Figure 5A–C and Figure 6A–C) when MYC decreased to 37.5% of that without THZ1 treatment. Marked cell death accompanied with the reduced number of cells in the G2 phase only occurred in the HepG2-MYC cells (Figure 5D–F and Figure 6D–F) after the MYC level was increased by 3.6-fold compared to that in the HepG2-LV cells with THZ1 treatment (lanes seven–eight vs. lanes three–four, Figure 8A(a),B(a)). With THZ1 treatment, only 18.5% of HepG2-LV cells were apoptotic when the MYC level was low, while 71.8% of the HepG2-MYC cells underwent apoptosis when the MYC level was high. Our data support the notion that exogenously overexpressed MYC confers the hypersensitivity of cancer cells arrested in the G2 phase to cell death with THZ1 treatment. 

Based on our observations that 1. overexpressed MYC promotes more cells’ entry into the active cell cycle, 2. cells in the G2 phase are highly susceptible to THZ1-induced cell death in HepG2-MYC cells, and 3. only elevated MYC and THZ1 treatment together markedly increase cleaved PARP and pH2AX (Figure 8A(b),A(c)), we argue that overexpressed MYC may have at least two distinct, yet conflicting, roles in cell proliferation and cell death: exogenously overexpressed MYC promotes cell entry into the active cell cycle for proliferation. However, differently from normal proliferating cells, the overexpressed MYC-driven entry of cells into the cell cycle causes these cells to be susceptible to DNA damage in the presence of THZ1. It was reported in doxorubicin-treated patient-derived neuroblastoma cells with a high level of MYCN that only cells in the G1 phase with repaired DNA damage may survive, while cells in other cell cycle phases eventually die [43], in line with our observations that the converged DNA damage response after high MYC-driven entry of cancer cells into the active cell cycle renders the hypersensitivity of HepG2-MYC cells to CDK7 inhibition. The diverged effects of suppressed and elevated levels of MYC on the entry of cancer cells into the cell cycle may also explain why the cancer cells with an elevated MYC level that is often linked to gene amplification or dysregulation are more sensitive to DNA-damage-induced apoptosis, although either a reduced or increased level of MYC was associated with apoptosis with treatment with THZ1. In HCC, highly expressed MYC is often the result of genomic amplification, which was detected in up to 70% of viral- and alcohol-related HCC, in 40–60% of early HCC [10,11]. In our study, we ectopically overexpressed MYC to mimic the dysregulated high MYC expression in cancer cells and investigated the sensitivity of high MYC-expressing cancer cells to CDK7 inhibition. MYC level in the HepG2-MYC cells was clearly increased compared to that in the HepG2-LV cells. As previously reported [44], we confirmed that THZ1 does not directly affect the regulation and functions of ectopically expressed MYC RNA and protein with a 3xFLAG-tagged MYC fusion protein.

Since the oncogenic transcription factor MYC is frequently dysregulated in human cancers and plays an important role in liver tumorigenesis [13,20,45,46], other approaches have been exploited to inhibit MYC activity. For example, bromodomain proteins such as BRD4 are critical for gene transcription. As a BRD4 inhibitor, JQ1 blocks the interaction between BRD4 and acetylated histones to suppress gene transcription, and has shown efficacy against many types of cancers [47,48,49,50]. In HCC, the inhibition of MYC is critical for JQ1 to suppress tumor growth [51]; JQ1 induces G0/G1 cell cycle arrest and enhances BIM expression to suppress HCC tumor cell growth [51,52]. The suppression of MYC has been found to be essential for MEK inhibitors to inhibit HCC cell proliferation and tumor growth [38]. The inhibition of MYC results in increased BIM protein, a proapoptotic protein of the Bcl-2 protein family that alone is sufficient to cause drug-induced apoptosis [53], supporting that MYC suppression causes apoptosis and inhibits tumor growth. Despite the shared similarities of MYC reduction and apoptosis between THZ1 and these inhibitors, THZ1-induced CDK7 inhibition exhibited several distinct features. In our study, we demonstrated that THZ1 strongly impeded the phosphorylation of RNAPII CTD in HCC cells. Different from JQ1-induced G0/G1 arrest, THZ1 caused cell cycle arrest at the G2/M phase, with increased pH2AX. These differences highlight the fact that the convergence of MYC-driven cell progression and THZ1-induced CDK7 inhibition led to an apoptotic DNA damage response, indicated by the increased pH2AX, resulting in the hypersensitivity for the apoptosis of MYC-driven cancer cells to THZ1.

In treating HCC, multikinase inhibitors, immunotherapy, and antiangiogenic monoclonal antibodies, along with other newly emerging therapeutics, have shown varied efficacy [6,7,8,9]. However, even combinatorial treatments of the therapeutics are limited to only a subset of the patients, and it is not well-defined whether the differential treatment responses are due to distinct interactions between immunity and cancer cells in the tumor microenvironment. In the case of CDK7 inhibition, our data support the notion that the MYC expression level may markedly impact the sensitivity of HCC cells to THZ1 treatment. It is thus predictive that HCC patients with high-MYC-expression tumors may exhibit a greater benefit from THZ1 treatment. In parallel, our data also suggest a limitation of THZ1 in treating HCC: tumors without high MYC expression are likely less sensitive. If genetic and/or epigenetic characteristics of cancer cells indeed determine treatment outcomes, then the development of a prognostic biomarker for each therapeutic would be a critical step to achieve precision medicine and maximal efficacy against HCC. 

Our observation that THZ1 induced DNA damage to promote the apoptosis of tumor cells and inhibit tumor growth has several potential implications. First, inhibitors targeting CDK7 may be evaluated in clinical trials for their efficacy in treating HCC. Indeed, a clinical trial has recently been initiated to examine whether CDK7 inhibition is effective in treating advanced solid tumors (https://clinicaltrials.gov (accessed on 18 March 2022); (NCT04247126). Second, THZ1 may be used in combination with other drugs such as sorafenib or regorafenib, two multikinase MAPK pathway inhibitors that target surface receptors and downstream kinases, to enhance the antitumor efficacy. Although sorafenib and regorafenib have been approved to treat HCC, they currently only exert moderate effects. It is known that THZ1 inhibits the expression of receptor tyrosine kinases, such as EGFR, in high-grade glioma. THZ1 has synergistic or additive effects when combined with the EGFR inhibitor erlotinib in breast cancer [54]. In HCC, the cooperation of EGF signaling and MYC has been shown to lead to aggressive cancer progression. A combination of THZ1 and an EGF/EGFR signaling pathway inhibitor may increase the efficacy in treating HCC. 

In summary, our findings indicate that targeting CDK7 with THZ1 may be a new plausible strategy to treat HCC, in which MYC plays crucial roles in cell proliferation and tumor growth. The strong apoptotic effects of THZ1 treatment may be further explored to achieve more effective treatments of HCC.

## 5. Conclusions

We demonstrated that the transcriptional vulnerability of HCC to THZ1 was not solely due to the effects of CDK7 inhibition. The hypersensitivity of HCC to THZ1 was conferred by the exquisite MYC-driven cell cycle dysregulation and apoptotic response due to transcription-inhibition-induced DNA damage. Our studies indicate that targeting CDK7 serves as a new plausible strategy for treating HCC, in which MYC plays crucial roles in cancer cell proliferation and tumor growth. 

## Data Availability

All data generated or analyzed during this study are included in this published article.

## Supplementary Materials

The following supporting information can be downloaded at: https://www.mdpi.com/article/10.3390/cancers14071714/s1, Figure S1: Dose-escalation effects of THZ1 on cell viability and proliferation in HCC cells; Figure S2: Construction of 3xFlag tagged MYC lentiviral expression vector and allele specific RT-qPCR analysis; Figure S3: Endogenous and lentiviral MYC protein expression; Figure S4: Allele specific MYC expression in HepG2-3xFlagMYC cells; Figure S5: MYC overexpression promotes cell cycle progression and alters response to THZ1; Figure S6: THZ1 decreases MYC (A) expression but increases the cleavage of PARP (B) in HepG2, Hep3B, and Sk-Hep-1 cells; Figure S7: THZ1 inhibits RNAII CTD phosphorylation in HepG2-LV and HepG2-MYC cells. Total protein extracts were prepared from the cells treated with vehicle or THZ1 (200 nM) for 3 h (A) or 48 h (B); Figure S8: THZ1 reduces MYC (A) and increases the cleavage of PARP (B) and pH2AX (C) in HepG2-LV as well as HepG2-MYC cells.
