# Supplementary material for: Converged DNA Damage Response Renders Human Hepatocellular Carcinoma Sensitive to CDK7 Inhibition
Source: Cancers (Basel). 2022 Mar 28;14(7):1714. doi: 10.3390/cancers14071714 (PMC8996977; doi:10.3390/cancers14071714)
Supplement: Supplementary file 1 [file cancers-14-01714-s001.zip › cancers-1635653-supplementary.pdf]

cancers

MDPI

Supplementary Materials: Converged DNA Damage Response Renders Human Hepatocellular Carcinoma Sensitive to CDK7 Inhibition

Guiqin Xie, Ailin Zhu and Xinbin Gu

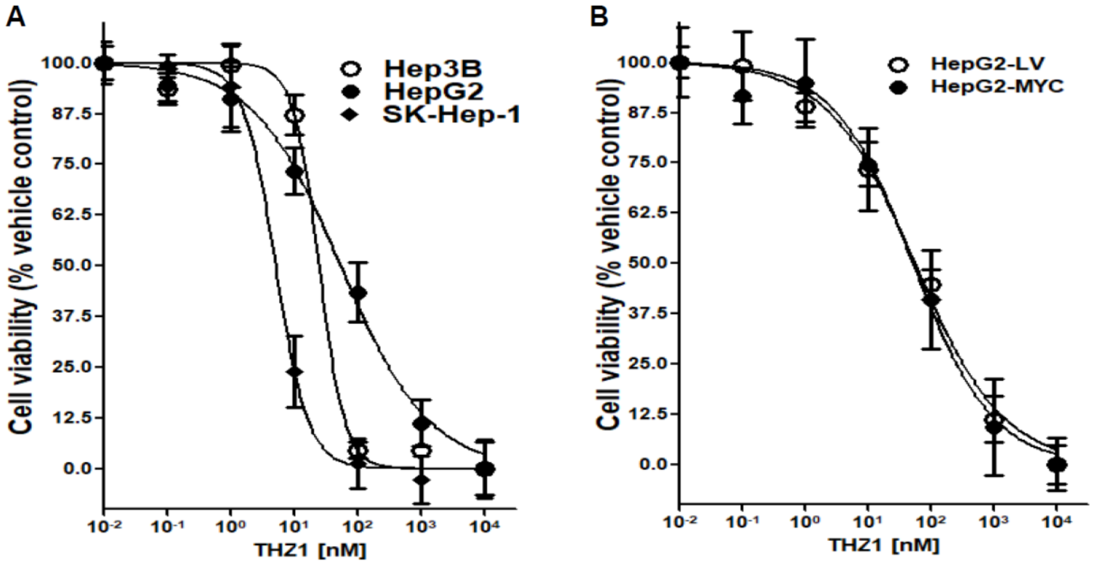

Figure S1. Dose-escalation effects of THZ1 on cell viability and proliferation in HCC cells. (A) Hep3B, HepG2 and Sk-hep-1 cells were exposed to THZ1 at the indicated doses for 5 days. (B) HepG2-LV and HepG2-MYC cells were exposed to THZ1 at the indicated doses for 5 days. Viability data of three independent experiments are shown as mean ± SEM (n = 3).

A

No.	Oligo	Sequence
1	3FMYCF1	CAGGCACCATGGGAGACTACAAGGACCACGACGGCGATTATAAGGATCACGACATCGACT
2	3FMYCR1	TCCAGGCTGGCGCCCTTGTCATCGTCGTCTTTGTAGTCGATGTCGTGATCCTTATAATC
3	3FMYCF2	ACGATGACAAGGGCGCCAGCCTGGATTTTTTTCGGGTAGTGGAAAACCAGCCTCCCGCGA
4	3FMYCR2	CGGGCCCTCTAGATTACGCACAAGAGTTCCGTAGCTGT

B

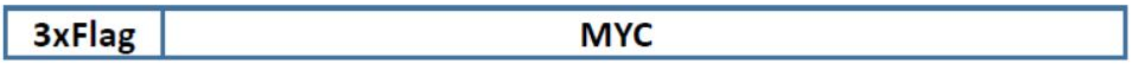

C

No.	Oligo	Sequence
1	MYC3FallF	GGACCACGACGGCGATTATA
2	MYC3FallR	CTGCTGCTGCTGGTAGAAGT
3	MYCendoAllF	CTGCCAGGACCCGCTTCTC
4	MYCendoallR	TGCTGCTGCTGCTGGTAGAA

Figure S2. Construction of 3xFlag tagged MYC lentiviral expression vector and allele specific RT-qPCR analysis. (A) Oligonucleotides were used to construct 3xFlag MYC lentiviral expression vector. After PCR assembly, the 3xFlag MYC DNA fragment was cloned into the NcoI and XbaI sites of the pHAGE-ERBB2 vector (Addgene #116734) to construct a pHAGE-3xFlagMYC vector and validated by the Sanger DNA sequencing analysis. (B) A schematic representation of the constructed 3xFlag MYC gene with a 3xFlag tag at the 5′ end of the gene. The 293FT cells were transfected with the pHAGE-3xFlagMYC vector and lentiviral packaging plasmids (Addgene #8454 and #8455) to make lentivirus. HepG2 cells were infected with the lentivirus to establish the HepG2-3xFlagMYC cell line. (C) Oligonucleotides were used to analyze allele specific endogenous *MYC* (MYCendoAllF and MYCendoallR) and 3xFlag tagged *MYC* (MYC3FallF and MYC3FallR) RNA expression. .

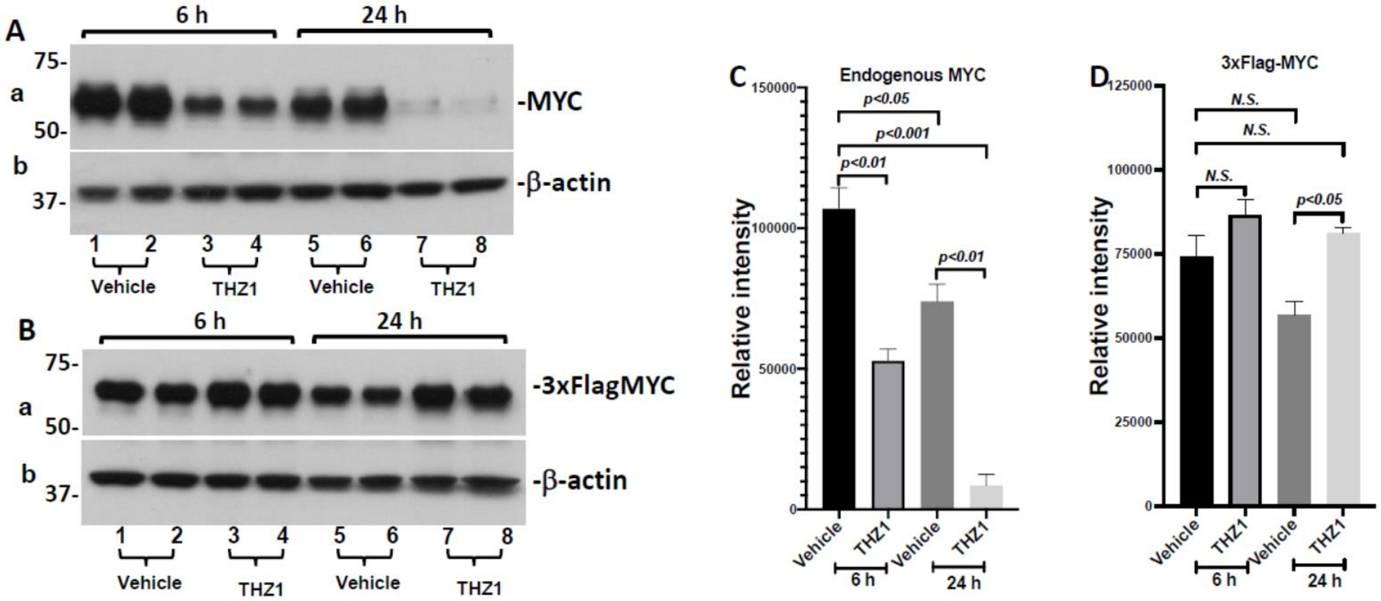

Figure S3. Endogenous and lentiviral MYC protein expression. (A) THZ1 reduces MYC protein expression in HepG2-LV cells. Total protein extracts were prepared from the cells treated with vehicle or THZ1 (200 nM) for 6 or 24 hr. Western blot analysis was performed for MYC (a) and β-actin (b). (B) THZ1 does not reduce 3xFlag tagged MYC protein expression in HepG2-3xFlagMYC cells. Total protein extracts were prepared from the cells treated with vehicle or THZ1 (200 nM) for 6 or 24 hr. Western blot analysis was performed for 3xFlagMYC with an anti-Flag antibody (a) and β-actin (b). (C, D) Band intensities in Fig. S4A and S4B were quantified for comparisons between vehicle-treated cells and THZ1-treated cells.

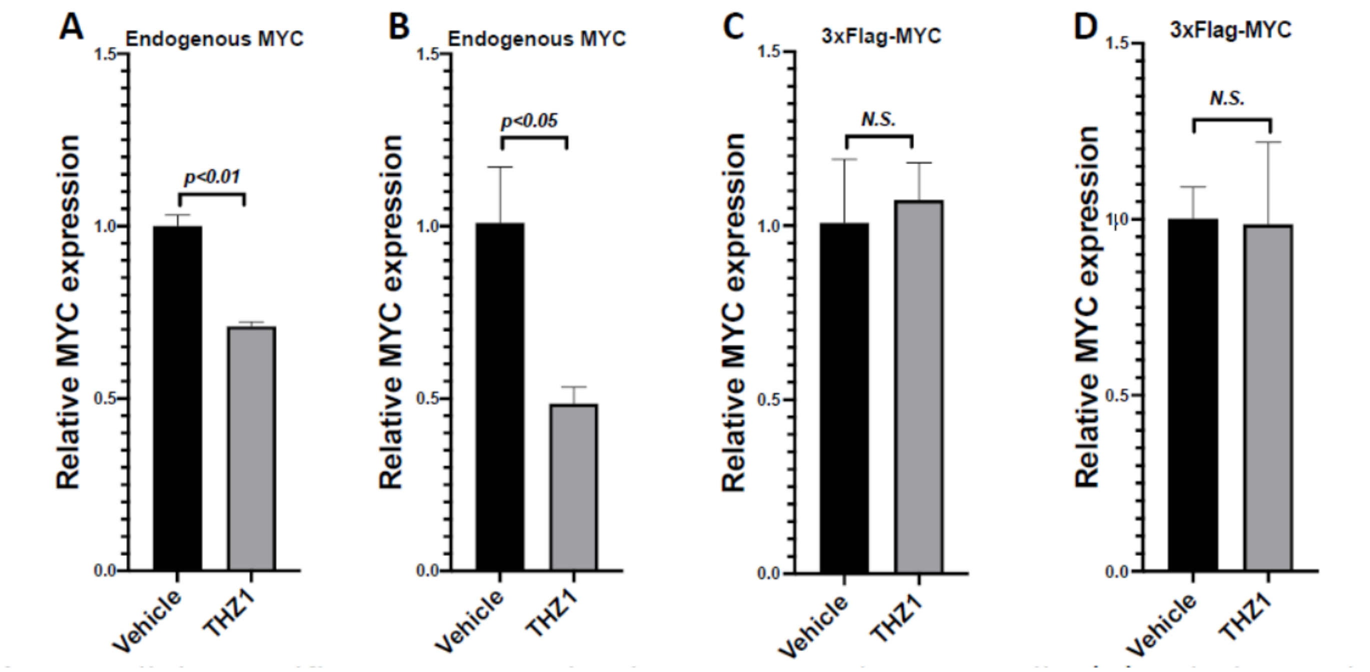

Figure S4. Allele specific *MYC* expression in HepG2-3xFlagMYC cells. (A) Relative endogenous *MYC* RNA expression after treatment with vehicle or TZH1 for 6 hr. (B) Relative endogenous *MYC* RNA expression after treatment with vehicle or TZH1 for 24 hr. (C) Relative lentiviral 3xFalg *MYC* RNA expression after treatment with vehicle or TZH1 for 6 hr. (D) Relative lentiviral 3xFlag *MYC* RNA expression after treatment with vehicle or TZH1 for 24 hr. The MYCendoAllF and MYCendoallR primers were used to perform RT-qPCR for endogenous *MYC* RNA expression while the MYC3FallF and MYC3FallR primers were used to perform RT-qPCR for lentiviral 3xFlagMYC *RNA* expression. Data of three independent experiments are presented as mean ± SEM (*n*= 3).

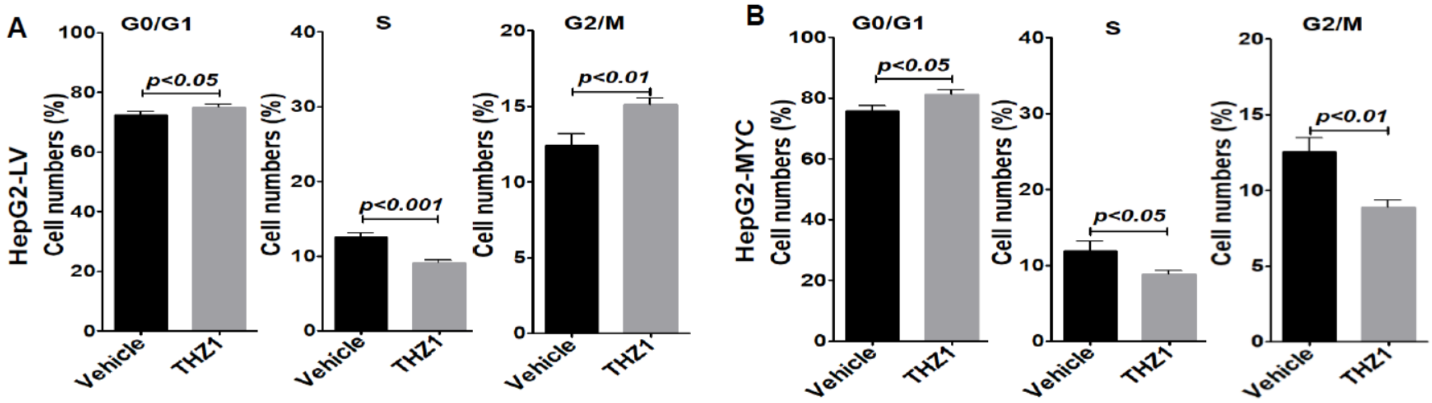

Figure S5. MYC overexpression promotes cell cycle progression and alters response to THZ1. HepG2-LV (A) and HepG2-MYC (B) cells cells were treated with vehicle or THZ1 (200 nM) for 24 hr. Flow cytometric analysis of cell cycle phases was carried out after DAPI staining. Data of three independent experiments are presented as mean ± SEM (*n*= 3).

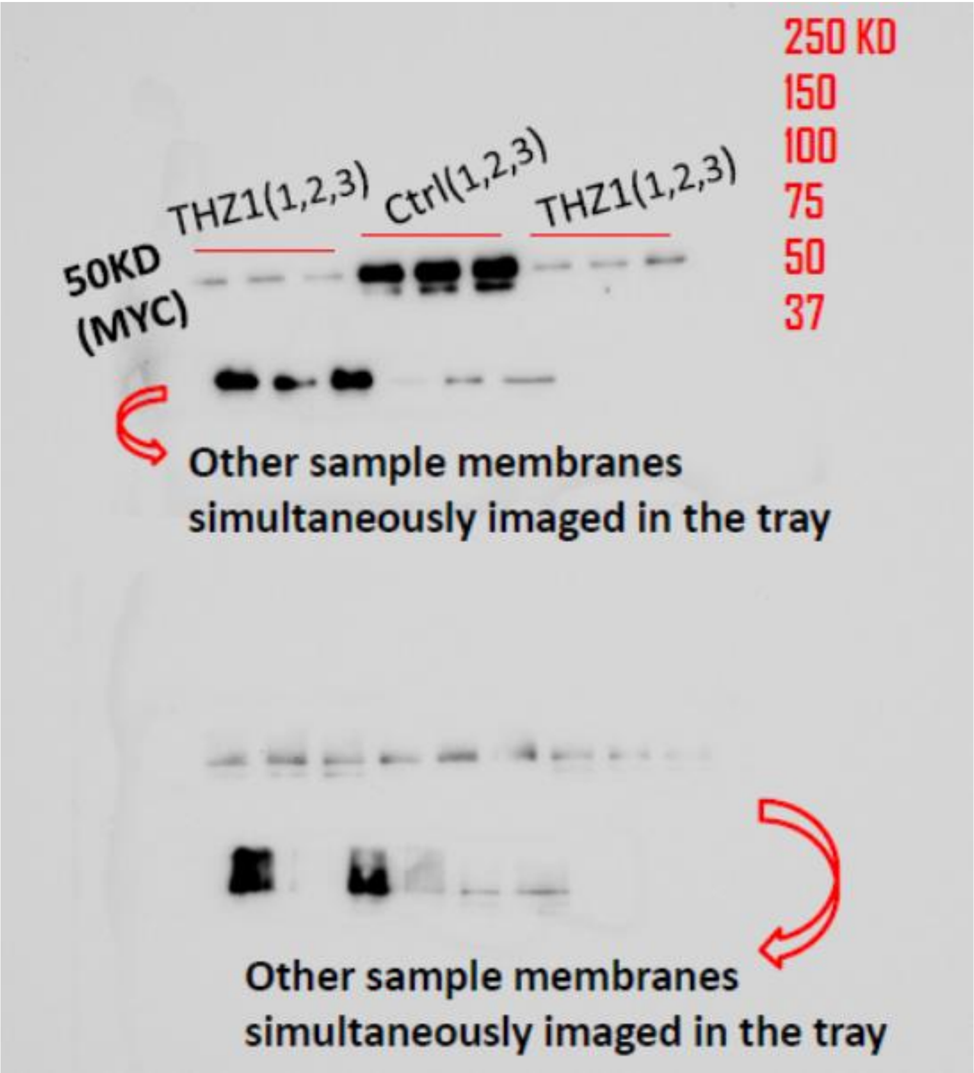

Figure S6. A HepG2_MYC .

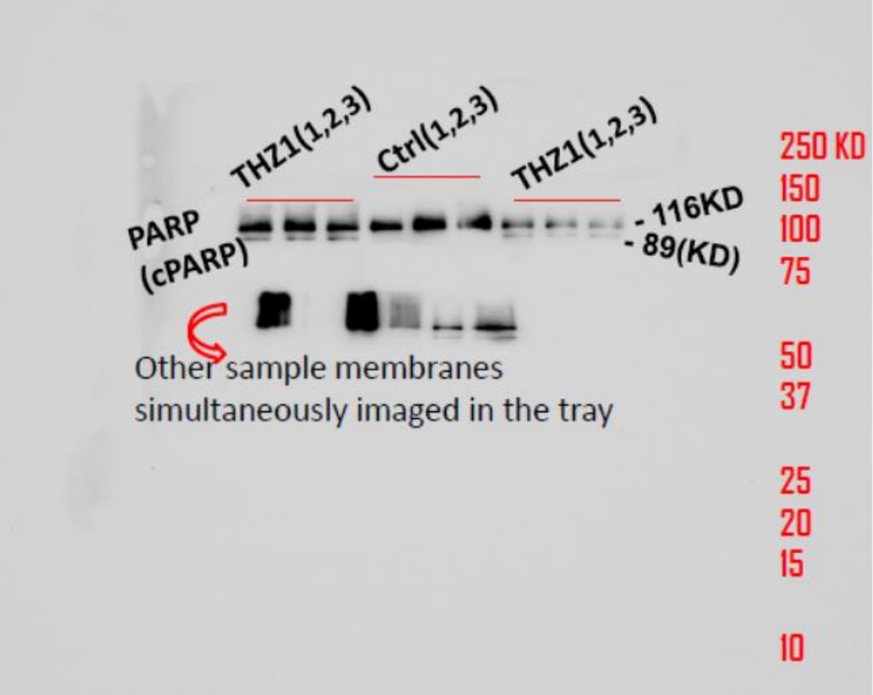

Figure S6. A HepG2_PARP

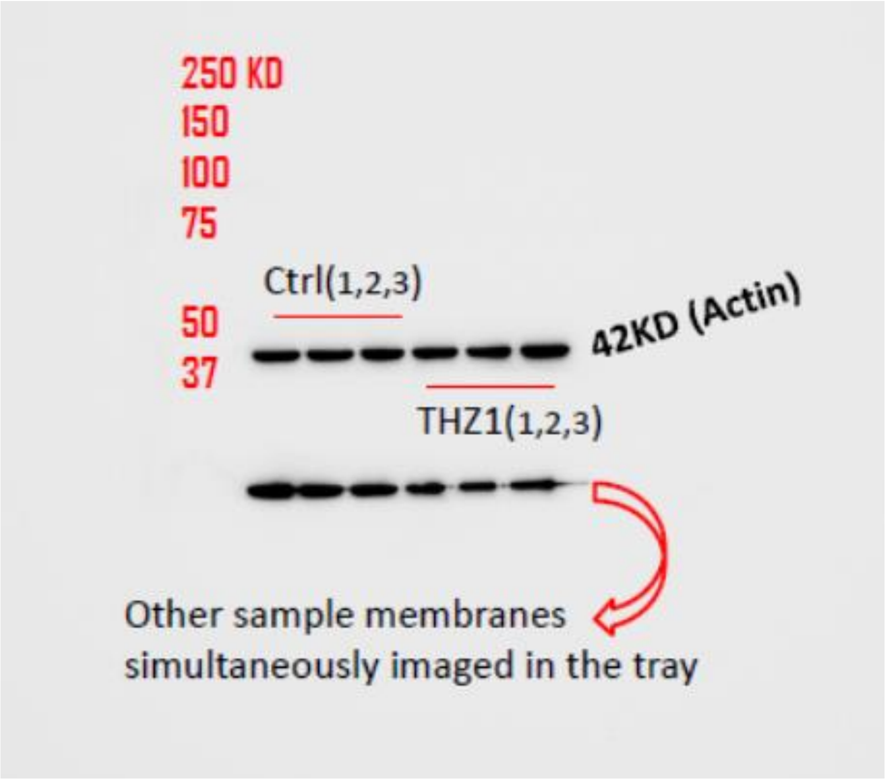

Figure S6. A HepG2_ Actin

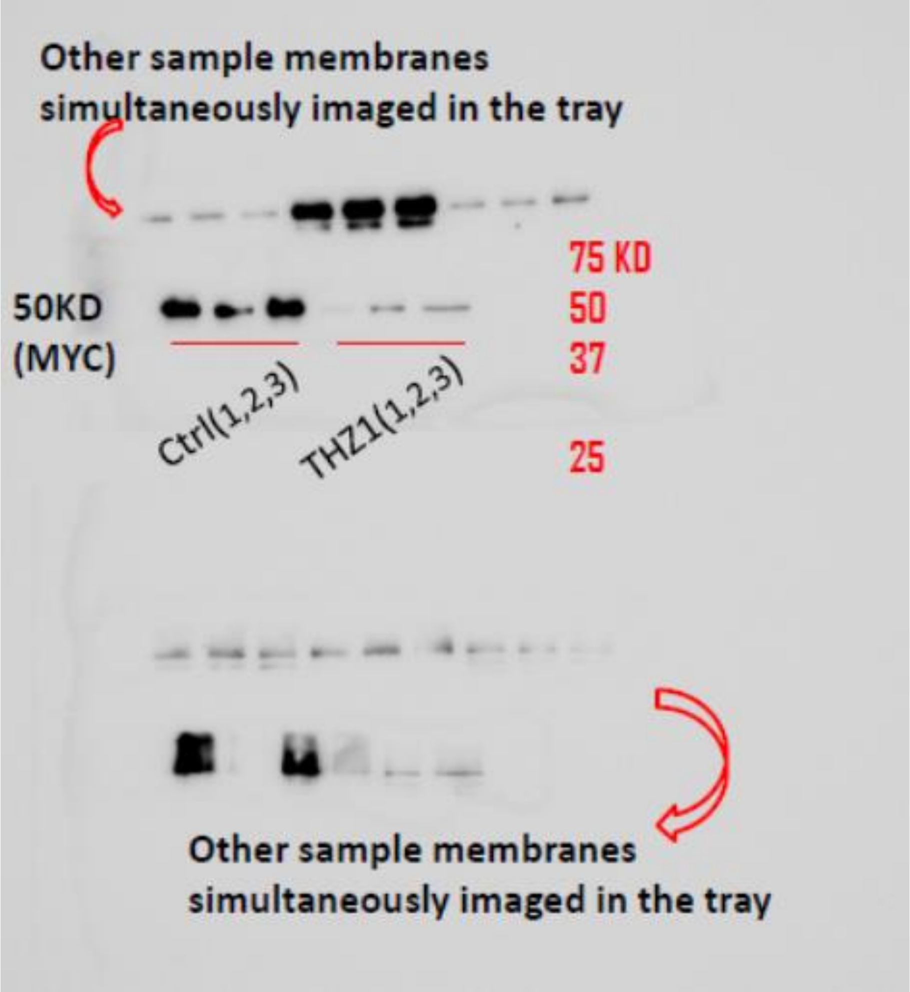

Figure S6. B Hep3B_MYC.

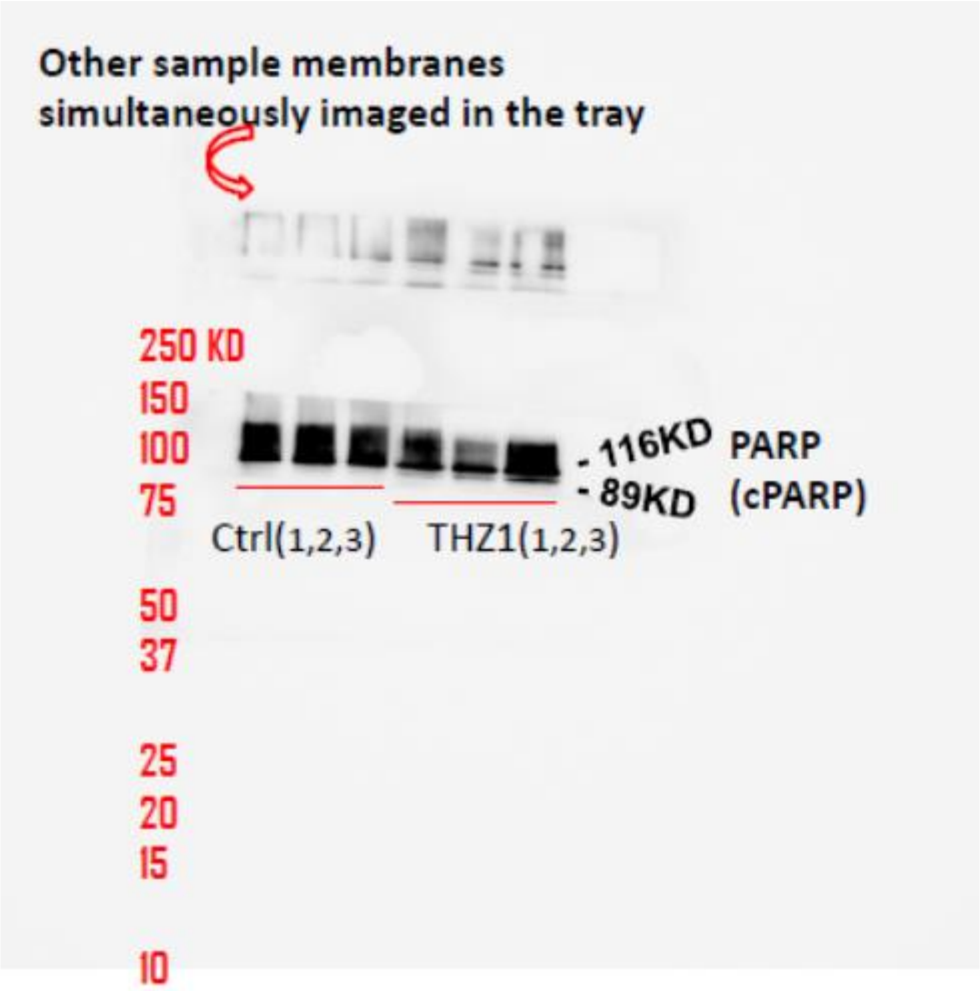

Figure S6. B Hep3B_PARP.

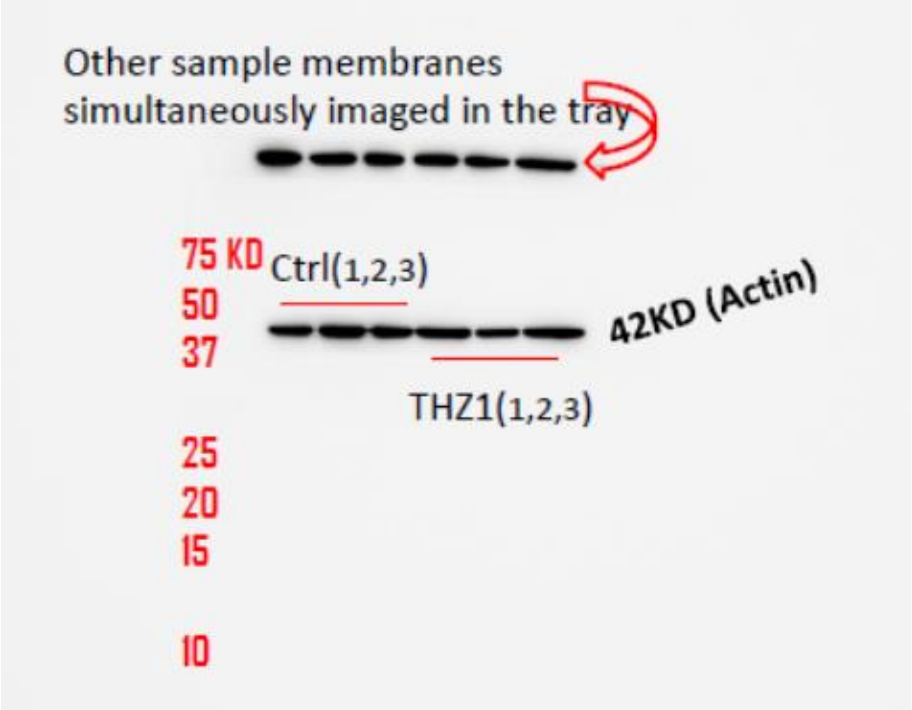

Figure S6. B Hep3B_ Actin.

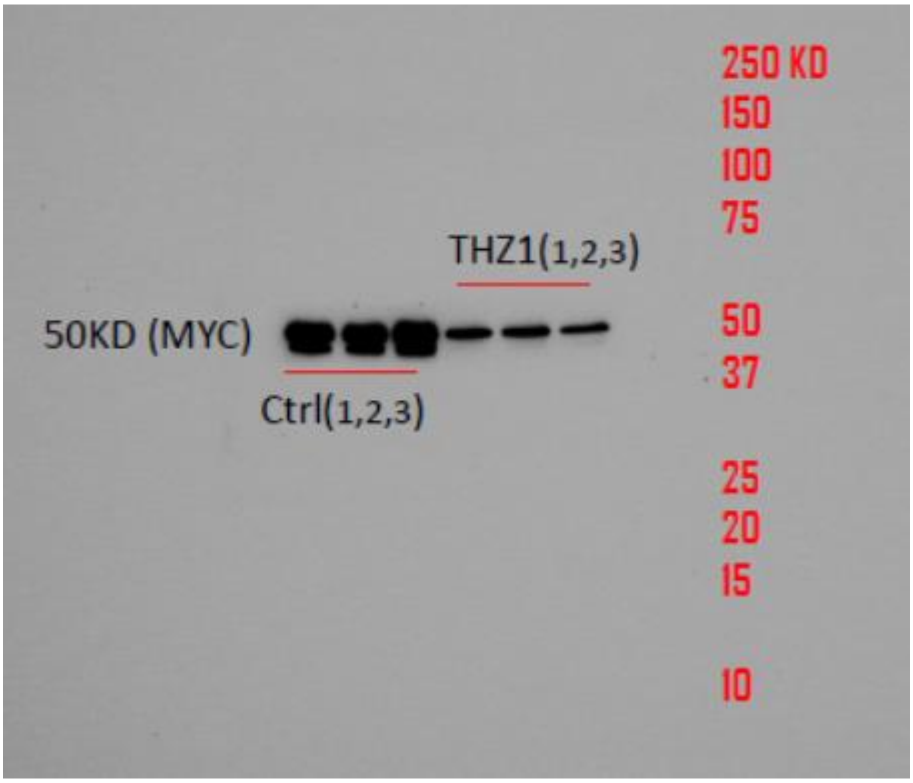

Figure S6. C SK-Hep-1_MYC.

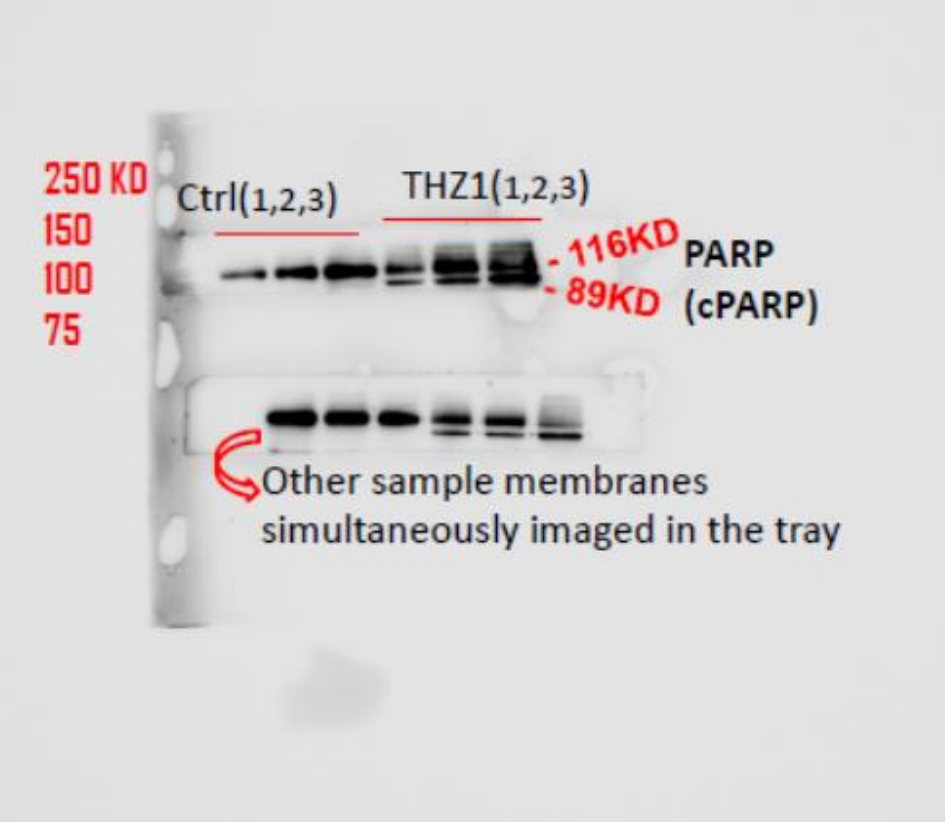

Figure S6. C SK-Hep-1_PARP.

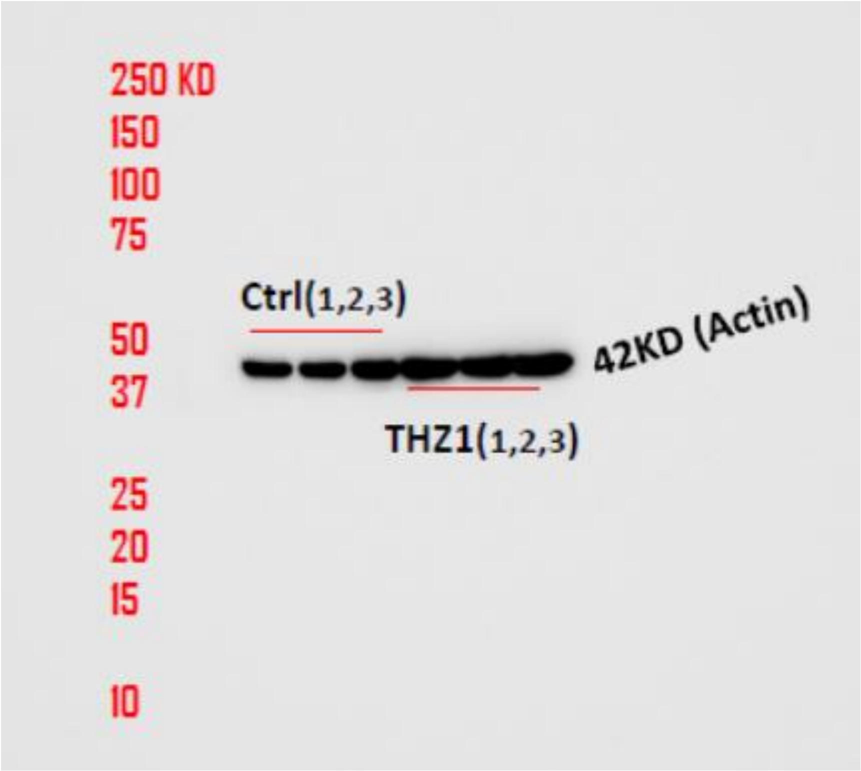

Figure S6. C SK-Hep-1_ Actin.

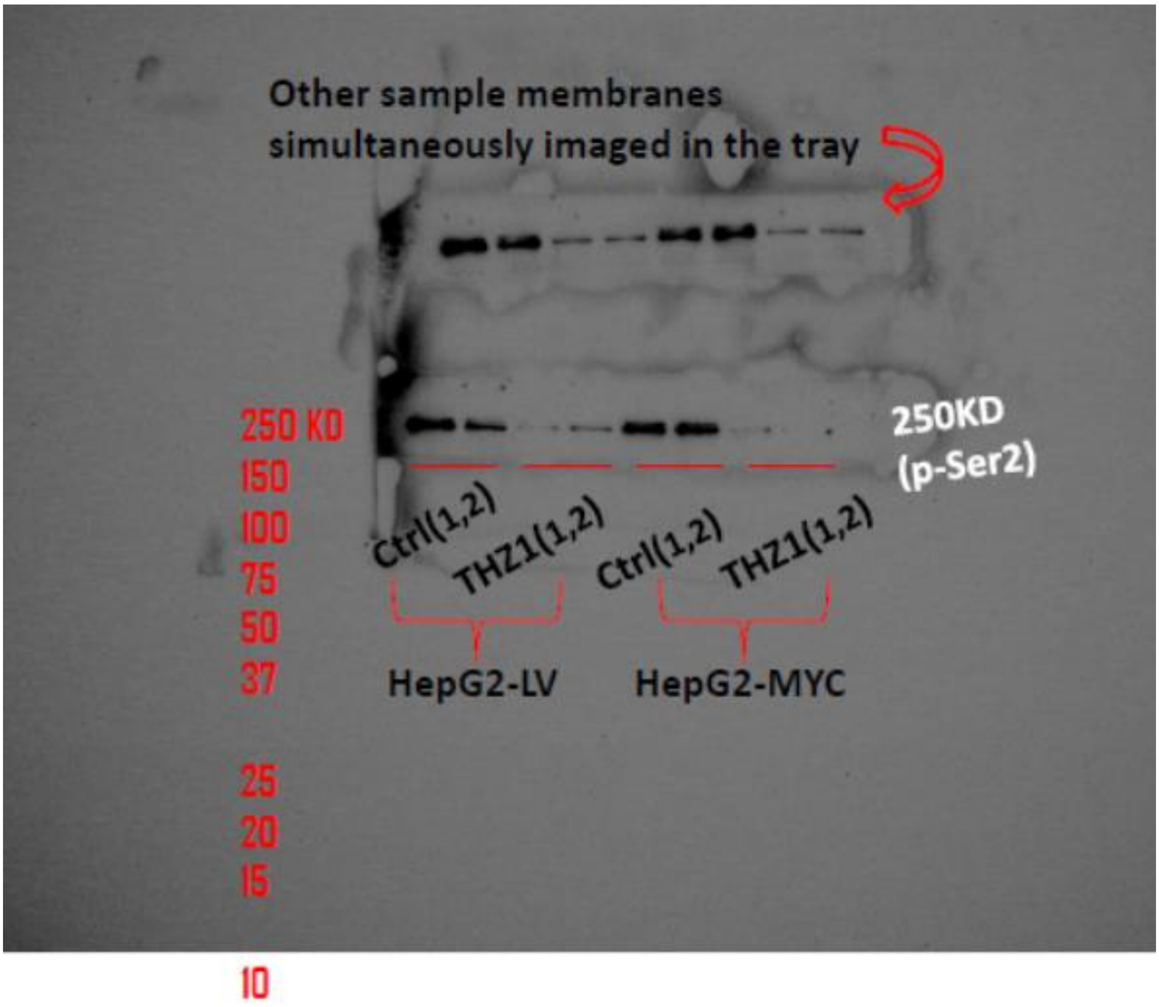

Figure S7. A HepG2-LV/MYC_ p-Ser2_THZ1 (3h).

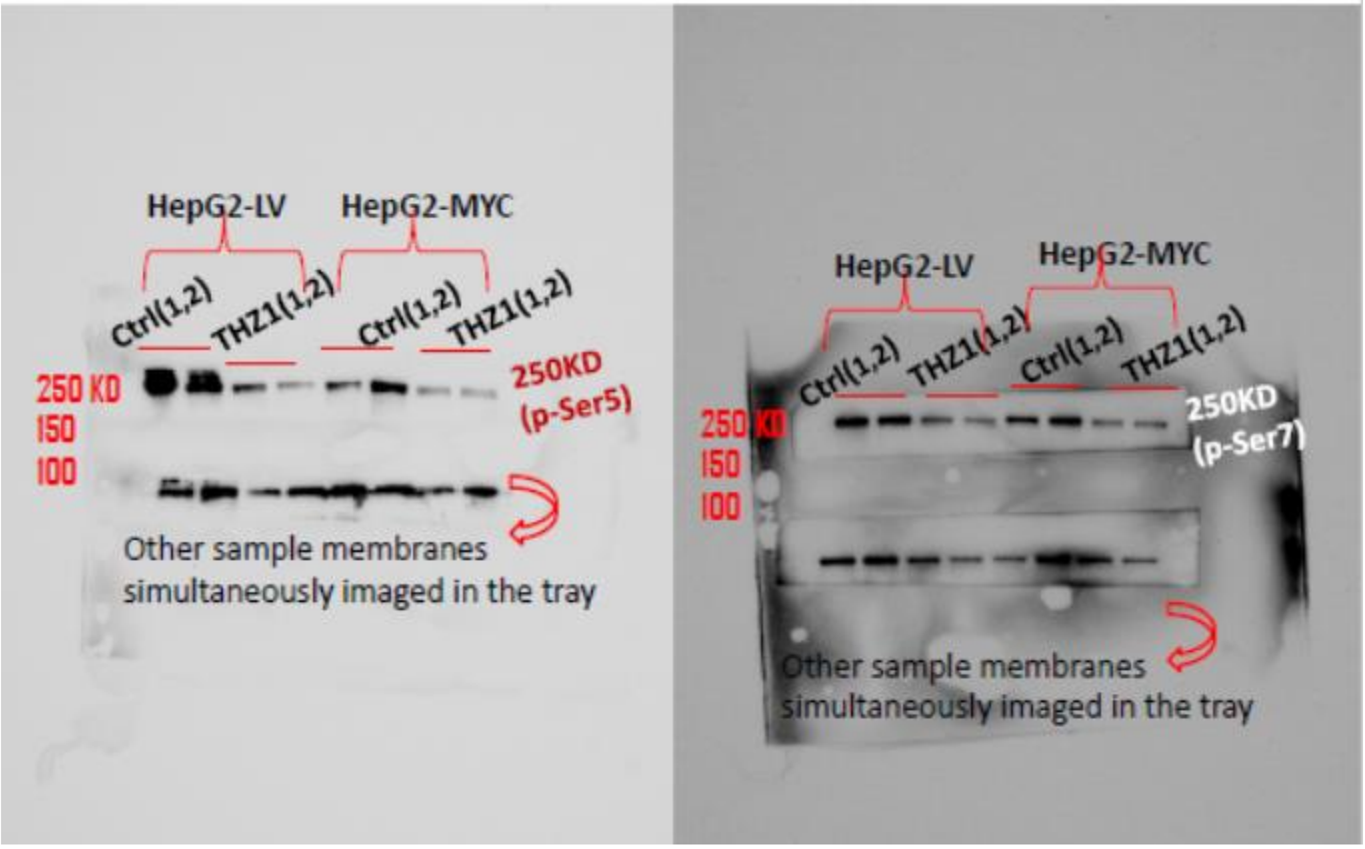

Figure S7. A HepG2-LV/MYC_ p-Ser5,7_THZ1 (3h).

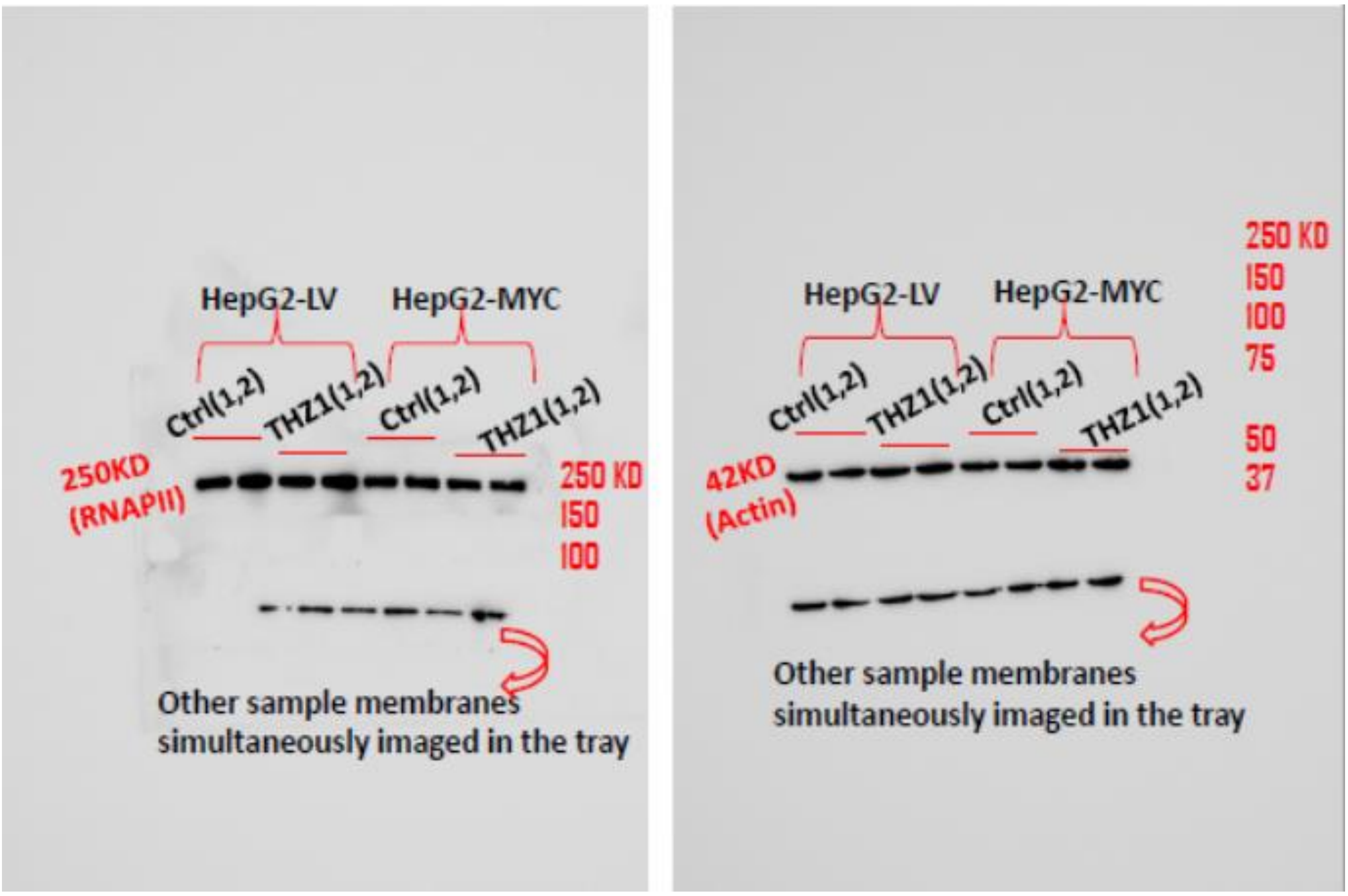

Figure S7. A HepG2-LV/MYC_ RNPII,Actin_THZ1 (3h).

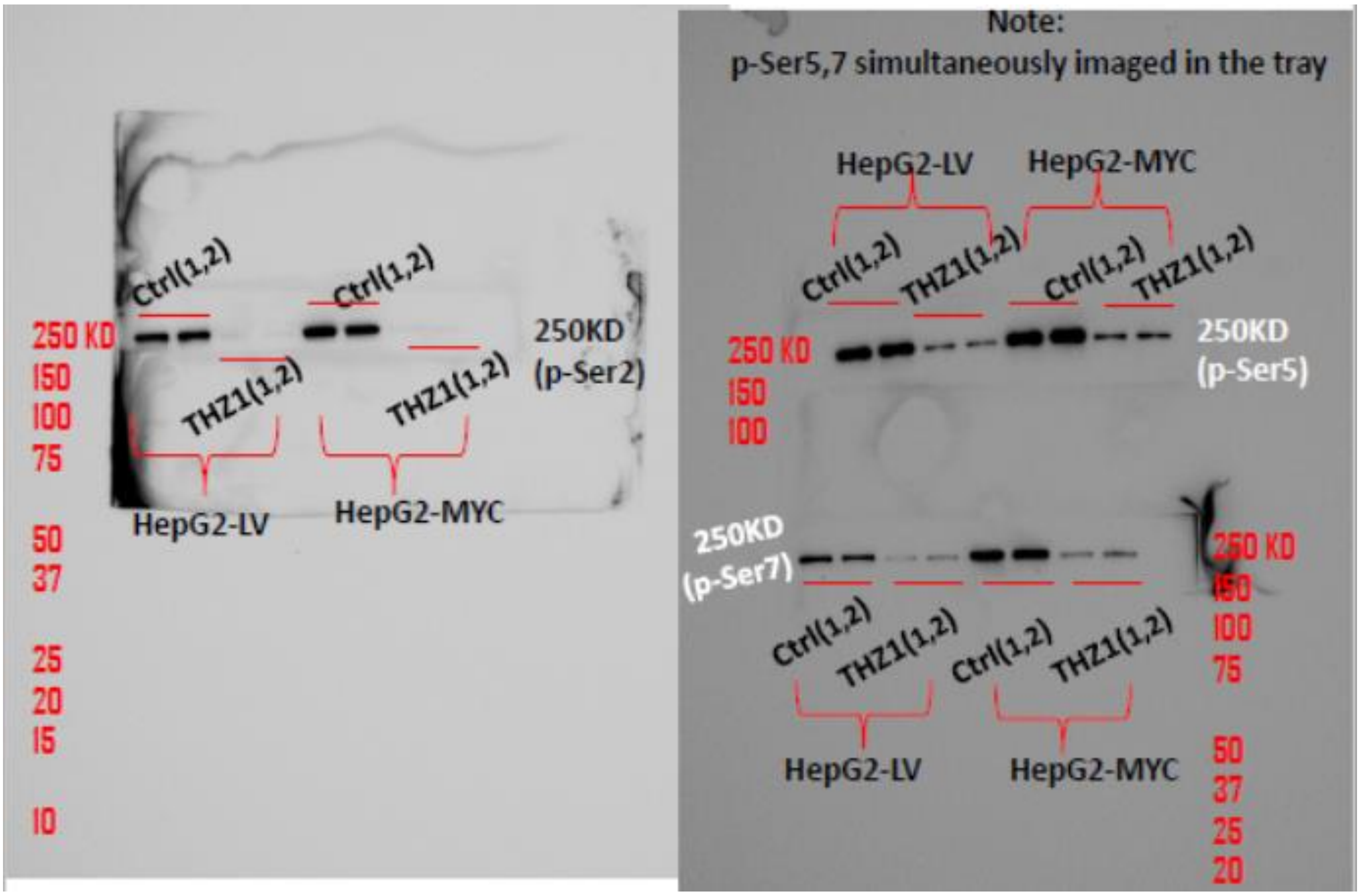

Figure S7. B HepG2-LV/MYC_ p-Ser2,5,7_THZ1 (48h).

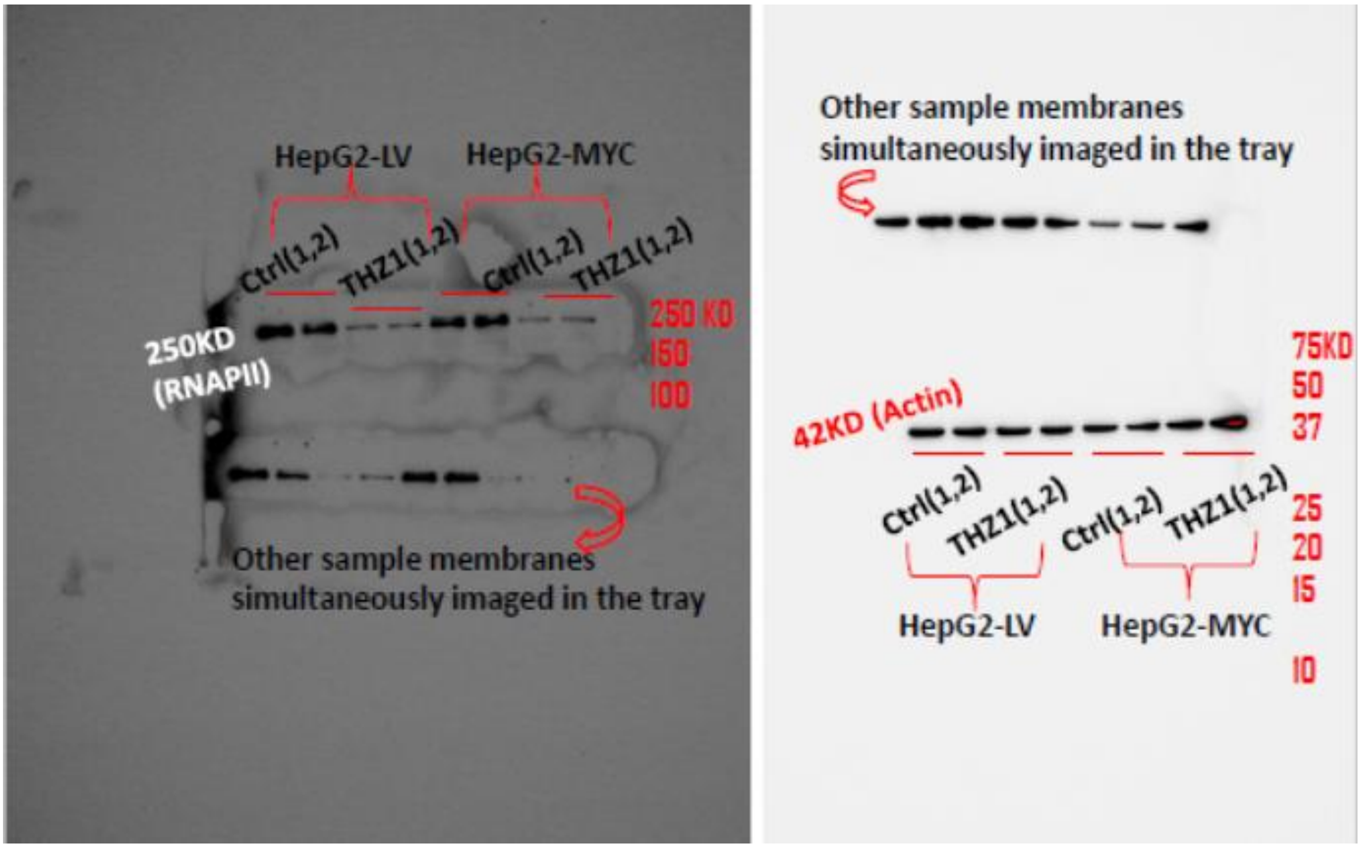

Figure S7. B HepG2-LV/MYC_ RNPII,Actin_THZ1 (48h).

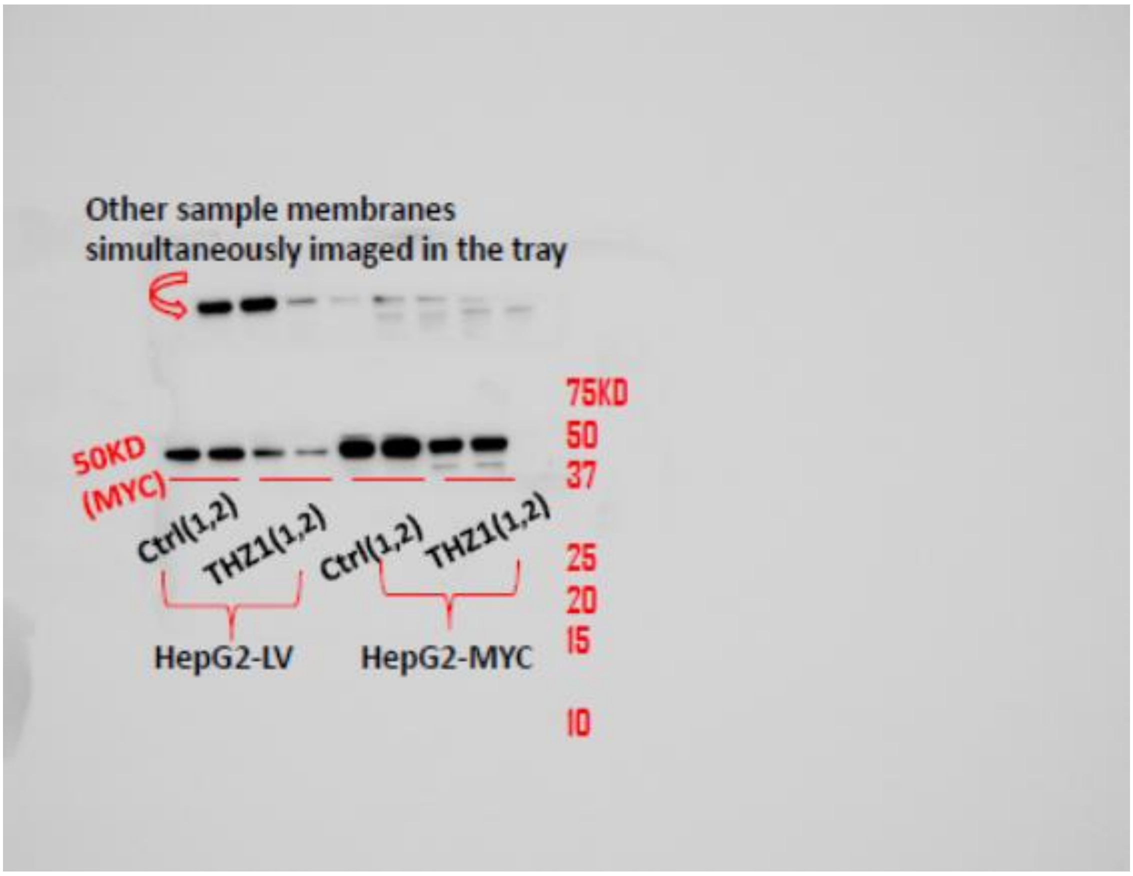

Figure S8. A HepG2-LV/MYC_ (MYC).

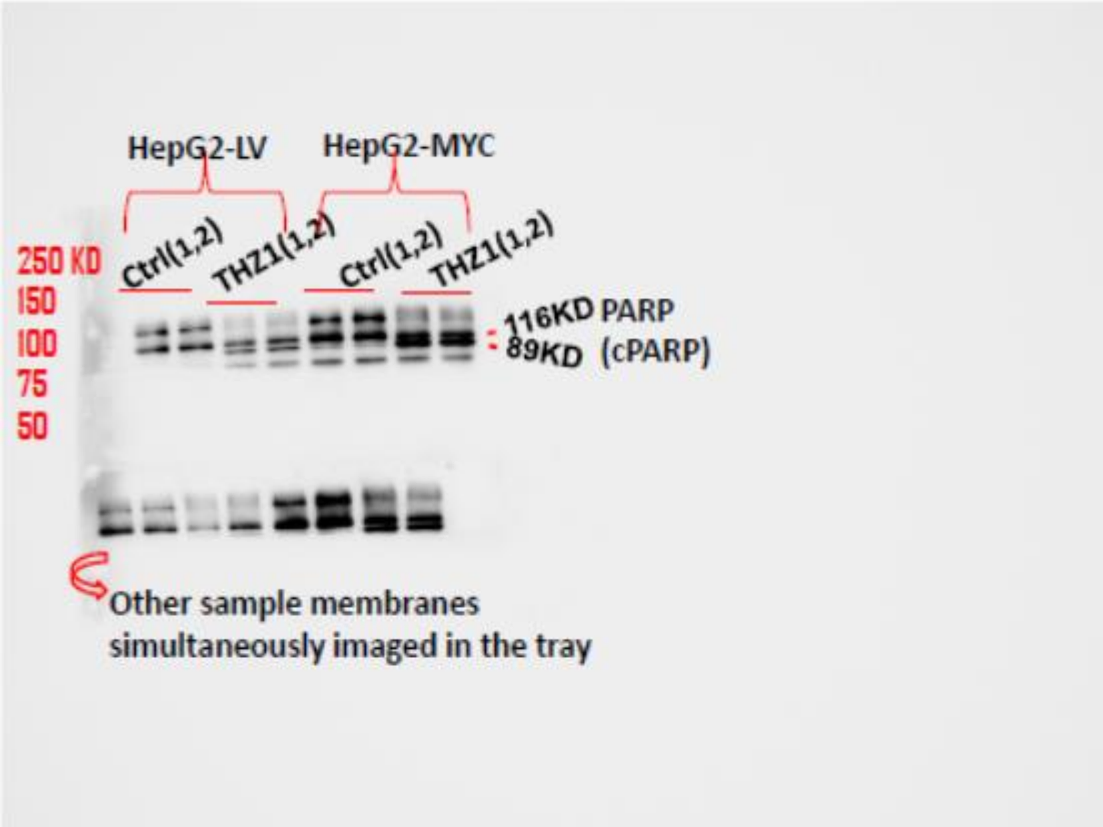

Figure S8. B HepG2-LV/MYC_ (PARP).

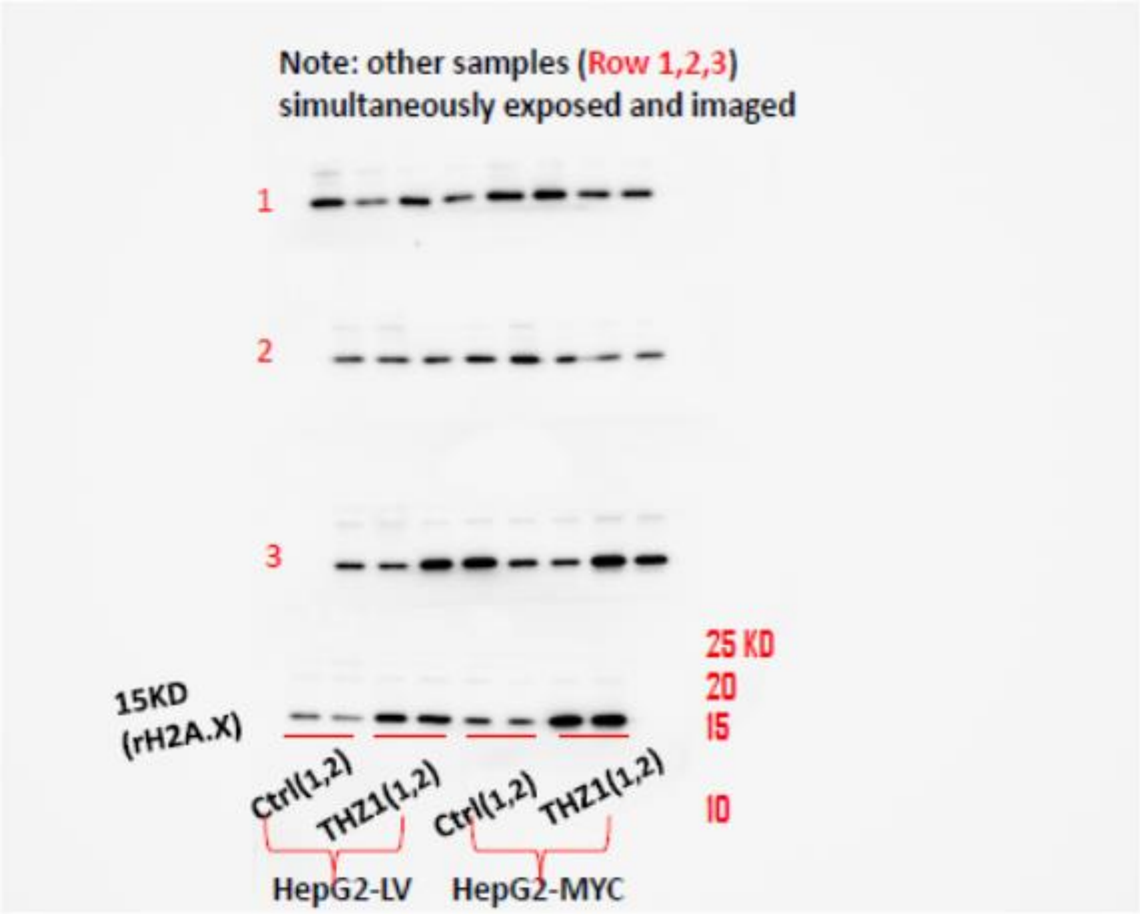

Figure S8. C HepG2-LV/MYC_ (pH2AX).

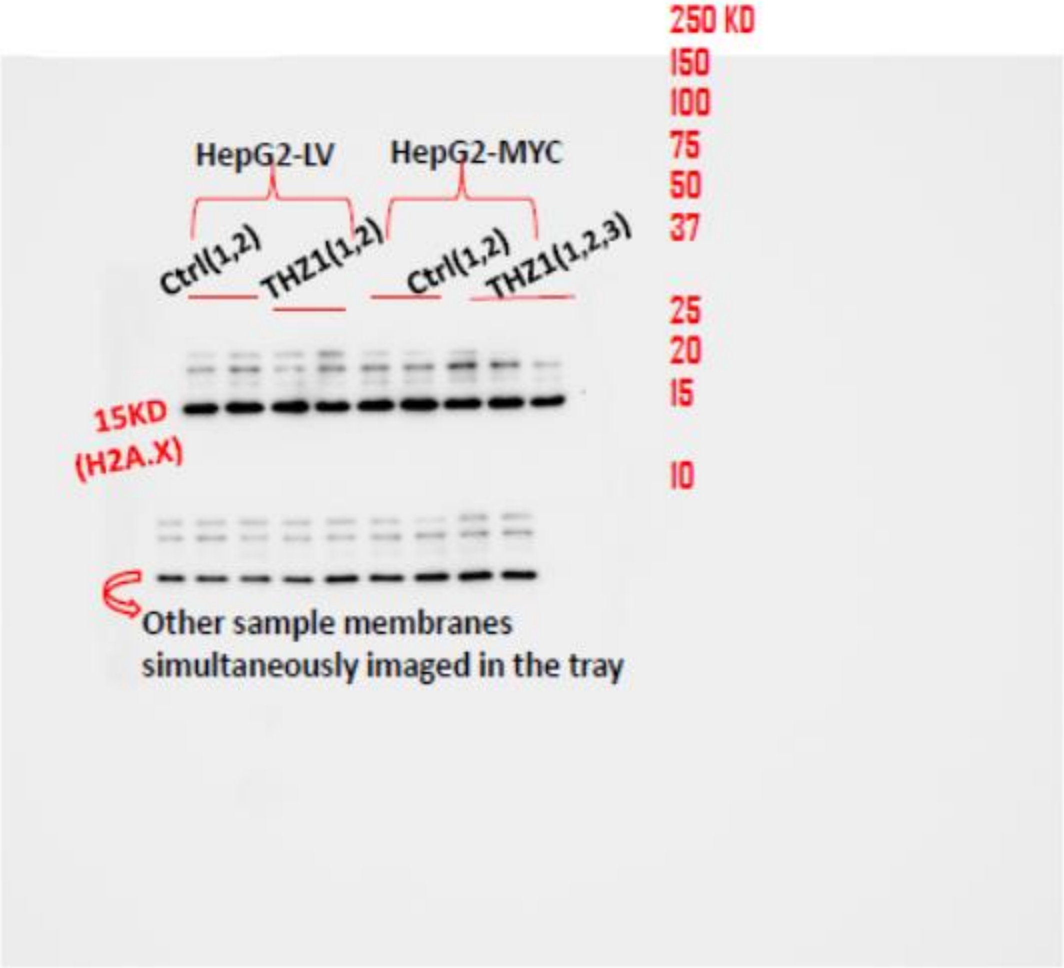

Figure S8. C HepG2-LV/MYC_ (H2AX).

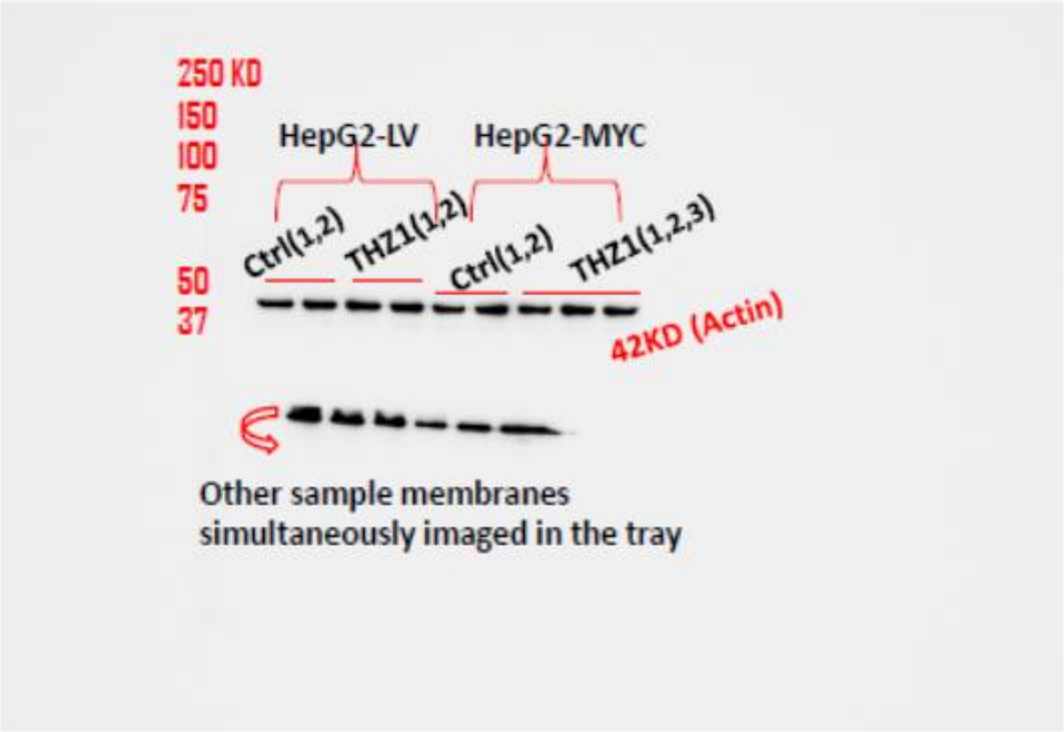

Figure S8. HepG2-LV/MYC_ (Actin) .
